# Non-clinical Psychosocial Mental Health Support Programmes for People with Diverse Language and Cultural Backgrounds: A Critical Rapid Review

**DOI:** 10.1007/s11013-024-09893-1

**Published:** 2025-01-29

**Authors:** Nathalia Costa, Rebecca Olson, Karime Mescouto, Jenny Setchell, Stefanie Plage, Tinashe Dune, Jennifer Creese, Sameera Suleman, Rita Prasad-ildes, Zheng Yen Ng

**Affiliations:** 1 School of Health and Rehabilitation Sciences, The University of Queensland, Brisbane, Queensland Australia; 2 School of Social Science, The University of Queensland, Brisbane, Queensland Australia; 3 The University of Queensland cLinical TRials cApability Team (ULTRA TEAM), Centre for Clinical Research, The University of Queensland, Brisbane, Queensland Australia; 4 RECOVER Injury Research Centre, The University of Queensland, Brisbane, Queensland Australia; 5 Australian College of Applied Psychology, Sydney, New South Wales Australia; 6 University of Leicester, George Davies Centre, Leicester, UK; 7 World Wellness Group, Brisbane, Queensland Australia; 8 Queensland Aphasia Research Centre, School of Health and Rehabilitation Sciences, The University of Queensland, Brisbane, Queensland Australia; 9 Surgical Treatment and Rehabilitation Service (STARS) Education and Research Alliance, The University of Queensland and Metro North Health, Brisbane, Queensland Australia

**Keywords:** Rapid review, Critical review, CALD, Non-clinical psychosocial support, Mental health

## Abstract

Low accessibility to mainstream psychosocial services disadvantages culturally and linguistically diverse (CALD) populations, resulting in delayed care and high rates of unsupported psychological distress. Non-clinical interventions may play an important role in improving accessibility to psychosocial support, but what characterises best practice in this space remains unclear. This critical rapid review addressed this gap by searching for, and critically analysing, existing research on non-clinical psychosocial support services, drawing from a critical realist framework and Brossard and Chandler’s (Brossard and Chandler, Explaining mental illness: Sociological perspectives, Bristol University Press, 2022) taxonomy of positions on culture and mental health. We searched PubMed, PsycInfo, LILACS, Scopus and Sociological Abstracts to identify non-clinical psychosocial support interventions for first-generation immigrant CALD populations delivered by lay-health workers. Thirty-eight studies were included: 10 quantitative, 7 mixed-methods and 21 qualitative. Most studies were conducted in North America (*n* = 19) and Europe (*n* = 7), with few conducted in low-income countries (Tanzania and Lebanon, *n* = 3 each, Kenya [*n* = 1]). Studies often focussed on specific interventions (e.g. psychoeducation) for targeted populations (e.g. refugees, Latinx immigrants); multimodal interventions (e.g. psychological support and food distribution) for broad populations were less common. Thirty-five different outcome scales were identified across quantitative and mixed-methods studies, with most covering depression, stress and trauma. Most studies identified significant improvements for at least one psychosocial outcome despite interventions being relatively short in sessions. Findings from qualitative studies highlighted varied engagement with theory-informed models of service, and identified important barriers to non-clinical psychosocial support services, including precarious resourcing. Our analysis suggests most studies were underpinned by split-relativist frameworks and focussed on interventions aimed at helping clients navigate the eurocentricity and complexity of mainstream services. Recognising the eurocentrism of universalist frameworks, working from a culturally relativist position, prioritising social determinants of health and using models that centre clients, flexibility, context, culture and community are likely to ensure best practice for non-clinical psychosocial support interventions.

**Supplementary Information:**

The online version contains supplementary material available at 10.1007/s11013-024-09893-1.

## Introduction

The percentage of immigrant populations are high in many high-income countries (e.g. 30% in Australia, 26.8% in New Zealand, 21.3% in Canada) and low- and middle-income countries (LMIC) (e.g. 33.4% in Jordan and 20.4% in Israel) (Organisation for Economic Co-operation and Development [OECD], 2022). The World Health Organisation urges healthcare systems to attend to the mental wellbeing of immigrants (WHO Issues Fact Sheet [WHO], 2023), as their resettlement in a new country can exacerbate or trigger mental health concerns, depending on the circumstances of departure (Chimienti et al., 2019; Lane et al., 2018; WHO, 2023) and the context of their adoptive countries (Mesa-Vieira et al., 2022; Naffi & Davidson, 2016). While migration itself is a unique process for each person, people undergoing immigration and seeking refuge often experience challenges when resettling in their host countries, including the uncertainties surrounding residence permits (Hvidtfeldt et al., 2020), new languages and social norms (Murray et al., 2010; Pottie et al., 2011), shifts in social and economic status, lack of interpersonal and institutional supports, discrimination and social exclusion (Fuller-Thomson et al., 2011; Hynie, 2018; Rezaei et al., 2023; Verelst et al., 2022). When these systemic stressors are not addressed, the effectiveness of mental health care interventions can be limited (Tay & Silove, 2017). Other structural factors such as marginalisation, lack of prioritisation in policies, funding, and low accessibility to affordable and culturally safe mental health services (Levesque et al., 2013; Stewart et al., 2011) further contribute to these challenges. People undergoing immigration and seeking refuge are often excluded from basic services in transitional countries, including mental health services (Ventevogel, 2014). Notably, mistrust of governance and service systems may add complexity to how these populations navigate mental health services and healthcare more broadly (Boukpessi et al., 2021; Ellis et al., 2011). In considering these findings together, it is not surprising that first-generation migrants, often referred to as culturally and linguistically diverse (CALD), or more recently culturally and racially marginalised (CARM) (Diversity Council Australia, 2023), are underserved in mental health settings (Chiu et al., 2018; González et al., 2010).

Before further discussing psychosocial mental health support for CALD groups, we first acknowledge the ongoing debate about the term CALD—a label that draws attention to linguistic, cultural and ethnic characteristics (Pham et al., 2021), but that is too broad to reflect the varying experiences of different communities (Pham et al., 2021; Sawrikar & Katz, 2009; Shepherd et al., 2021). By foregrounding culture while grouping all minority groups together, the term homogenises unique cultures and undermines acknowledgement of cultural diversity (Sawrikar & Katz, 2009). It has also been argued that the term positions the white/English-speaking group as the default, othering non-white and non-English-speaking individuals (Sawrikar & Katz, 2009; Shepherd et al., 2021). While this term can be problematic, it is widely used in the literature we reviewed. Therefore, we prioritised this term in identifying relevant studies, and use the term CALD to refer to cultural or linguistic minorities (in number or power) who originally came from different countries and spoke languages distinct from the language spoken in the host country.

Under-servicing CALD communities often results in missed opportunities for mental health promotion and early intervention, delayed care and high rates of unsupported psychological distress (Blignault et al., 2022). Constituting a form of health inequity, low accessibility to psychosocial support services^1^ disadvantages such communities (Braveman & Gruskin, 2003). Importantly, accessibility to support services is not enough to meet the needs of CALD communities. Some suggest such services also need to encompass cultural competencies (i.e. being cognisant of cultural factors that shape approaches to mental health) and structural competencies (i.e. understanding of how social, political and economic factors influence the production, experience and maintenance of the presenting health issue) (Salam et al., 2022). Others argue that competency is not enough; services must be culturally safe (Curtis et al., 2019) and responsive (Kirmayer & Jarvis, 2019). Culturally safe means that healthcare practitioners, healthcare organisations and health systems need to be prepared to critique the ‘taken for granted’ power structures and be prepared to challenge their own culture and cultural systems rather than becoming ‘competent’ in the culture of others (Curtis et al., 2019). Culturally responsive indicates that strategies are taken to address culture and context in health services, e.g. involving an interpreter or cultural brokers (Kirmayer & Jarvis, 2019).

Brossard and Chandler’s (2022) taxonomy of positions on culture and mental health usefully informs critical engagement with existing debates. In this taxonomy, those coming from the *universalist position* see mental health disorders as singular and universal. While culture is understood to influence how mental disorders manifest, from this position the core of what mental illness is remains the same. The *split-relativist position* sees some mental health disorders as universal and others as culturally specific. The *radical relativist position* aligns best with the approach of new critical ethno-psychiatry (Gaines, 1992), where the conceptualisation and experience of mental disorders are seen and treated as culturally dependent (i.e. the way people think about and experience them is culturally dependent), although it acknowledges the need to avoid biomedical essentialising of culture (Sargent & Larchanché, 2011). It also critically considers this through the lens of Galtung’s (1969) and Farmer’s (2004) frameworks of structural violence, where policies of entitlement and exclusion equally determine both health vulnerability and accessibility of healthcare, and Galtung’s further-developed conceptualisations of cultural violence (1990), where dominant categories are a product and imposition of Western psychiatry (Brossard & Chandler, 2022). This is particularly true in the case of former imperial (e.g. UK, Europe) and settler-colonial (e.g. Australia, USA, Canada, New Zealand) contexts where colonialism, neo-colonialism and cultural stereotypes deeply inculcated, not only in psychiatric models, but in psychopathologies and social determinants of health for ethnic minorities (Beneduce, 2019; Fanon, 1963; Fassin & Rechtman, 2005). Support provided by cultural peers may transcend the epistemic imposition of the universalistic position that underpins delivery of care within most western healthcare systems. Indeed, findings suggest support provided by peers can be more effective than that provided by healthcare professionals, as the latter can exacerbate feelings of oppression and the former may be more culturally safe and responsive (Loumpa, 2012).

Non-clinical organisations and interventions delivered by lay-health workers (LHWs) can be potentially perceived as less stigmatising (Lai et al., 2020; Loumpa, 2012), given the intense stigma associated with seeking clinical care for mental health in many non-western cultures (Lam et al., 2010; Park et al., 2018; Vonnahme et al., 2015). Therefore, non-clinical organisations and interventions can play an important role in fostering the integration of individuals from CALD backgrounds into their host communities by promoting access to psychosocial mental health services and fostering information sharing across settings and sectors (Ratnayake et al., 2022). Here, we define non-clinical psychosocial mental health support as interventions and services delivered by LHWs (Barnett et al., 2021). LHWs are defined as ‘nonprofessional providers, who have shared lived experiences with the individuals they serve’ but are not health professionals, who often work as bridges to formal services or navigators through such services (Barnett et al., 2021). Growing evidence of the effectiveness of non-clinical interventions underpins the World Health Organizations’ recent support of such practices in low- and middle-income countries (Keynejad et al., 2021). However, to the best of our knowledge, the literature on non-clinical psychosocial support for people from CALD backgrounds has not been reviewed to date. For instance, Ratnayake et al. (2022) reviewed immigrant settlement organisations which provide support to these populations, but the authors did not focus on mental health services (Ratnayake et al., 2022). They also limited their inclusion criteria to organisations and health systems from high-income countries. These contextual limitations open avenues for further exploration since LHWs may work outside these organisations, and LMIC host a substantial (76% as of 2022) proportion of CALD immigrants (United Nations High Council for Refugees, 2023).

To generate guidance for government and non-government organisations to better support CALD communities with non-clinical psychosocial services for people from CALD backgrounds, we conducted a critical rapid review to answer the following question: what characteristics, practices and contexts of non-clinical psychosocial mental health support generate what outcomes for immigrants with CALD backgrounds? By addressing this research question, we aimed to offer insights to policy makers and non-government organisations regarding how to attend to the needs of these populations when making decisions about funding, service design and delivery, while considering the realities of challenging contexts. Such insights will also be useful to other critical health scholars working with organisations to co-design and evaluate non-clinical psychosocial services for people from CALD backgrounds.

## Method

**Fig. 1:**
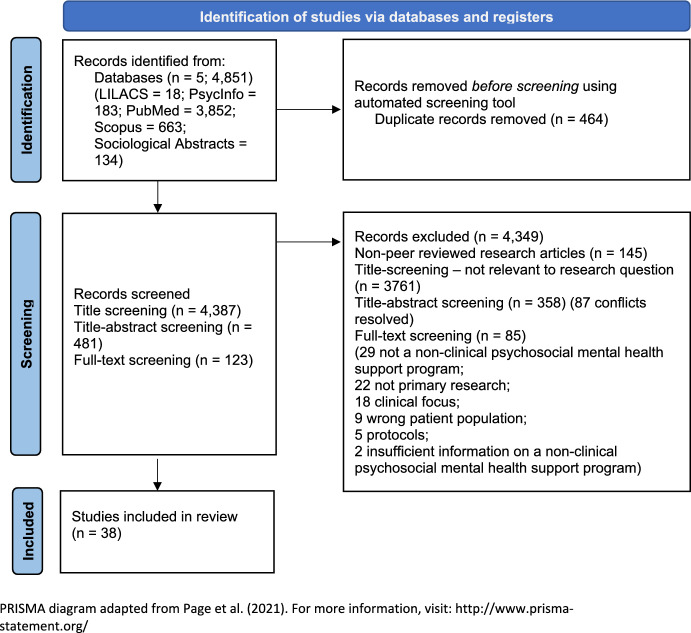
PRISMA flow diagram. PRISMA diagram adapted from Page et al. (2021). For more information, visit: http://www.prisma-statement.org/

We used the Preferred Reporting Items for Systematic Review and Meta-Analysis (PRISMA) (Fig. 1, Appendix B in electronic supplementary material) (Page et al., 2021). The review was not registered. We conducted a critical rapid review: an assessment of what is known about relevant practice while prioritising efficiency and using systematic review methods to search for and critically^2^ analyse existing research to arrive at a new interpretation or model (Grant & Booth, 2009). Of note, our multicultural team included seven researchers with expertise in health and social science, and three individuals who work in mental health services. Eight of us are first-generation immigrants, with relevance for our review process, including analysis and interpretation of findings. Specifically, we were able to leverage a fuller understanding of experiences with the healthcare system and beyond based on our positionality. Because we took a critical stance on the rapid review, the initial analytic observations and interpretations were shaped by our academic, clinical and personal experiences of privilege and oppression. For instance, when analysing some of mixed-methods and quantitative papers, NC experienced frustration given the mismatch between the problems the research sought to address and the interventions that followed (at times, these did not address systems of oppression). She was, therefore, aware that the content of the articles resonated with things she had observed living in Brazil and experienced as a migrant in Australia. As Charmaz (1990, 1999) and Braun and Clarke (2019) argue, such positionings and affective entanglements are central to interpretations.

## Eligibility Criteria

As we were interested in gaining a comprehensive understanding of the literature, we did not restrict our search to any specific designs (i.e. we were interested in quantitative, qualitative and mixed-methods studies). Therefore, we took a flexible approach to our eligibility criteria, drawing from PICO (population, intervention, comparison intervention, outcome measures) and PICo (population, phenomenon of interest, context) frameworks (Stern et al., 2014), detailed below. To maintain our focus on non-clinical psychosocial support for immigrants, we excluded clinical interventions delivered directly by clinicians, unless the clinician was doing the intervention with LHW to support the LHW themselves (e.g. offering them support to address the emotional and physical burden associated with humanitarian work) (Chemali et al., 2017). All studies reporting interventions delivered by clinicians were excluded. Aligned with the temporal constraints of a critical rapid review, we limited our scope to peer-reviewed primary research publications.

### Population

We included studies that focussed on one or more of the following populations: (1) CALD populations—specifically first-generation immigrants, refugees and people seeking asylum, including individuals, families and groups from minority (in number or power) cultural and language groups; (2) LHW providing psychosocial mental health support programmes for people with diverse language and cultural backgrounds (e.g. interpreters, case workers, volunteers); (3) Other stakeholders involved in providing psychosocial mental health support to CALD populations (e.g. administrative staff, service managers). Healthcare professionals were accepted as part of the population if they were working alongside LHWs to offer non-clinical psychosocial mental health support programmes for CALD immigrants, refugees and people seeking asylum. We excluded studies that reported on second-generation (and beyond) immigrants; Indigenous populations; populations whose cultural diversity is based in sexual orientation or gender identity and/or clinicians providing clinical mental health support directly to immigrants.

### Interventions or Phenomenon of Interest

We included studies which investigated non-clinical psychosocial mental health support programmes for CALD immigrants, refugees and people seeking asylum, non-clinical and clinical populations, including people with moderate to severe mental health diagnoses. As previously mentioned, we defined non-clinical psychosocial mental health support as interventions and services delivered by LHWs [i.e. people who have shared lived experiences with the individuals they serve, but are not health professionals (Barnett et al., 2021)]. The positioning of the person delivering and receiving the intervention is significant here, with the relationship between LHWs and participants of that of peers. As such, we excluded clinical psychosocial mental health support programmes delivered directly and exclusively by or for clinicians, using therapeutic techniques (e.g. CBT).

### Comparison Intervention

Any comparator was considered valid, meaning that we did not exclude any articles based on the comparator.

### Context

We included studies on interventions and practices conducted in community, clinical, educational and home settings, and diverse types of healthcare systems (e.g. by country and type: private/public). Studies conducted on practices within in-patient hospital-based care were excluded.

### Outcomes

Any measure of service provision including communication, information and education, assessment, intervention and other resources was included. Outcome measures related to changes in mental health and wellbeing (e.g. depression, anxiety, improvement of housing, improved access to food, improved education, admission rates, support satisfaction) for clients, families, communities, support workers and stakeholders were also considered.

## Search Strategy

The search strategy (see Appendix A) and the database selection were determined collaboratively as a team, in consultation with a librarian experienced in developing database searches for systematic reviews. Searches were conducted in July 2022, in the databases PubMed (including PubMed clinical queries), PsycInfo, LILACS, Scopus and Sociological Abstracts. We used indexes, subject headings and synonyms of key words. Where needed, as illustrated in Appendix A, database ‘AND’, ‘OR’ and ‘*’ functions, and different English spellings were also used to maximise search outcomes.

## Study selection

The search results were exported into EndNoteX9.0 (© 2022 Clarivate, United States), and duplicates were removed. Results were then uploaded to Covidence (© 2022 Covidence, Australia), a screening platform. One member of our team (ZN) then independently conducted the title screening, with any queries deliberated collectively. Two members (ZN and NC) then equally contributed to abstract screening. Subsequently, ZN screened all full-text publications, with NC and RO both acting as independent screeners of one half of these publications each, and arbiter (where they were not a screener) for any publications where there were disagreements. To capture a wide range of articles, publications in English and other languages in which the research team were fluent (i.e. Dutch, German, French, Portuguese, Spanish, Mandarin) were considered during the screening processes.

## Data extraction

After study selection, ZN extracted the following data from included studies: authors, year of publication, study design, location, study aims, intervention, who delivered the intervention, intervention duration, setting and number of settings where the intervention was delivered, language spoken by participants, participants’ cultural background, participants’ migration status (if available), outcomes considered and study findings. A second reviewer (NC for quantitative and mixed-methods studies, RO for qualitative studies) reviewed the data extraction and wrote analytical notes throughout the double-checking processes (see ‘comments’ columns in Tables 1, 2, and 3).

**Table 1:** Summary of the quantitative studies investigating psychosocial support programmes

Author (year) Study Design Location	Study aims	Intervention Who delivers the intervention (*n*) Intervention duration	Setting (*n*) Population (*n*)	Language Culture Migration status	Outcomes considered	Results^a^	Comments
Badger et al. (2020) Randomised controlled trial (comparator: telephone interpersonal counselling (TIPC) delivered by a social worker United States	To test two 2-month psychosocial interventions ([TIPC] and Supportive Health Education [SHE]) to improve QoL outcomes for Latinas with breast cancer and their informal caregivers	SHE: normal breast health and breast cancer, routine tests, treatments, side effects, lifestyle interventions, resources and referrals Bilingual bicultural paraprofessional (NR) 8 weeks	Telephone 230 dyads	English or Spanish Latinx NR	***Depression:*** 8-item short form from the PROMIS ***Stress:*** 10-item perceived stress scale ***Symptom distress:*** General Symptom Distress Scale ***Social support:*** 8-item PROMIS short forms for informational support, emotional support and social isolation	Latinx cancer survivors: At 2 months, TIPC produced lower depression scores compared to SHE (*p* = 0.04, 95% CI (0.01, 3.82)). At 4 months, SHE reduced symptom distress (*p* = 0.04, 95% CI (− 1.45, − 0.03)) and social isolation compared to TIPC (*p* = 0.03, 95% CI (− 4.27, − 0.24)). At 6 months, total number of symptoms was lower in SHE than in TIPC (*p* = < 0.01, 95% CI (− 1.97, − 00.58)). Caregivers: At 2 months, total number of symptoms (*p* = 0.04, 95% CI (− 1.29, − 0.04)), symptom distress (*p* = 0.05, 95% CI (− 1.52, − 0.01)) and anxiety (*p* = 0.01, 95% CI (− 5.38, − 0.66)) were lower, and self-efficacy for symptom management was higher in SHE compared to TIPC. Caregiver depression was lower in TIPC compared to SHE at 4 months (*p* = 0.04, 95% CI (0.11, 4.30)).	All materials for the study were translated in Spanish using the translation-back-translation method. After translation, a bilingual bicultural research staff member audited the translation with suggestions for revisions. Five Latinx cancer survivors provided feedback and changes were made based on the comments provided. The interventions were tailored to cultural values and beliefs of the participants (e.g. importance of family and close friends, displaying mutual respect, valuing personal relationships, being polite on the telephone and in all interactions, a relationship of trust). Most survivors were living in poverty (73% had a household income below $30,000 a year)
Cardeli et al. (2020) Pre-post study United States	(1) to describe the psychosocial needs of resettled Bhutanese refugee students; (2) to evaluate the impact of skill-based groups on these students’ sense of school belonging and mental health	Trauma Systems Therapy Skill-based group A Bhutanese cultural broker and a mental health clinician (one each [implied]) 12 weeks (1 h in length)	School (1) Bhutanese Refugee Youth (34)	Bhutanese Bhutanese Refugees	***Aim 1*** ***Traumatic exposure***: War Trauma Screening Scale (WTSS); Child Version of the UCLA PTSD Reaction Index (UCLA PTSD-RI) for DSM-IV ***Acculturation:*** Language, Identity, and Behaviour scale (LIB) ***Aim 2*** ***PTSD:*** UCLA PTSD-RI DSM-IV ***Depression:*** Depression Self-Rating Scale for Children (DSRS-C) ***School belonging***: The Psychological Sense of School Membership (PSSM) Scale	No significant difference between PTSD symptom scores from baseline (mean = 21.48, SD = 15.20) to follow-up (mean = 20.00, SD = 11.44), *t*(26) = 0.60, *p* = 0.55. No significant difference between depressive symptoms from baseline (mean = 10.26, SD = 3.19) to follow-up (mean = 10.19, SD = 2.93), *t*(30) = 0.108, *p* = 0.92. No significant difference between psychological sense of school belonging from baseline (mean = 65.29, SD = 9.41) to follow-up (mean = 67.16, SD = 11.01, *t*(30) = − 1.01, *p* = 0.32)	Group sessions included games, role-plays, experimental exercises and cross-cultural discussions about similarities and differences between American and Bhutanese culture. Major themes of group sessions were teamwork, respect, communication, diversity, self-regulation and self-esteem
Greene et al. (2021) Feasibility trial (comparator: usual care [e.g. information about existing services]) Tanzania	To examine the relevance, acceptability and feasibility of evaluating a multi-sectoral integrated violence and mental health-focussed intervention (Nguvu)	A multi-sectorial integrated violence- and mental health-focussed group intervention A lay refugee incentive worker and a humanitarian partner (5 pairs) 8 weeks	Local women’s groups in a refugee camp (43) Congolese refugee women from Nyarugusu refugee camp in Tanzania (311)	Kiswahili Congolese Refugees	***Depression:*** Hopkins Symptom Checklist (HSCL-25) ***Anxiety:*** Hopkins Symptom Checklist (HSCL-25) ***Post-traumatic distress:*** Harvard Trauma Questionnaire (HTQ) ***Physical and sexual violence:*** Abuse Assessment Screen ***Intimate partner violence:*** Conflict Tactics Scales, demographic health surveys	Small and non- significant between-group differences for all outcomes considered, except for post-traumatic stress symptoms, which was lower in the intervention group relative to the control group (Mean Diff = − 0.22; 95% CI: − 0.43, − 0.01)	The intervention was developed amongst female refugees from the eastern Democratic Republic of the Congo to reduce intimate partner violence and distress, drawing from Cognitive Processing Therapy. The gender-based violence response programme that existed at the time of the study consisted of case management and referrals to protection, medical or legal services (aid services, education about women’s rights, arranging safe shelter)—women from both intervention and control groups were able to access these services. There were differences in the likelihood of participant intervention completion by facilitator pair suggesting possible heterogeneity in the quality of implementation across facilitators. Analyses were not powered to detect significant between-group differences in outcomes.
Im et al. (2018) Pre-post study Kenya	To explore the effect of a trauma-informed psychoeducation (TIPE) on both the mental health and psychosocial domains amongst Somali refugee youth	TIPE modules which promoted refugee resilience, alongside components of peace education, conflict resolution, management skills and problem-solving methods Youth Somali leaders (10) and community health counsellors (5) 12 sessions	Community-based organisation (1) Somali youth (145)	Somali Somali Refugees	***Trauma exposure:*** A 10-item list of refugee youth trauma developed based on a community needs assessment (war-related trauma and community violence trauma) ***PTSD:*** PTSD Check List-Civilian Version (PCL-C) ***Psychosocial factors:*** a 5-point Likert scale developed by community leaders, psychiatrists and counsellors, which assessed (1) attitudes towards violence, (2) sense of community, (3) social support, (4) emotional coping, (5) problem-solving and (6) awareness around mental health and psychosocial needs	No changes in PTSD symptoms when considering the total sample. For youth with no/low PTSD, there was a significant increase in score (27.42 [SD = 6.67] to 34.48 [SD = 12.83], *p* < 0.001), indicating a possible increase in self-awareness of symptoms, whereas those with high PTSD reported lower scores (50.09 [SD = 7.52] to 31.93 [SD = 13.86], *p* < 0.001). There were significant improvements in sense of community (6.83 [SD = 3.69] to 8.19 [SD = 2.71], *p* < 0.001), social support (4.29 [SD = 2.56] to 5.04 [SD = 2.10], *p* < 0.05) and awareness (6.87 [SD = 3.79] to 7.76 [SD = 3.75], *p* < 0.05).	TIPE sessions included education on multifaceted impacts of trauma on the body, mind, social relationships and spirituality, followed by psychosocial competencies, such as emotional coping and problem-solving, community and support systems, and conflict management skills Taken together, the results on PTSD symptoms might indicate that PTSD symptoms are already present in the majority of refugee youth, whether minor or severe, but low awareness in trauma responses likely hinders recognition and acceptance of trauma responses until exposure to psychoeducation. The authors acknowledge that adopting a PTSD score as a measure of outcome is a limitation of the study, as it is underpinned by a Western concept. The authors acknowledge the need to consider other factors that tend to impact on the mental health of refugees (e.g. poverty, poor livelihood, discrimination, family violence)
Lai et al. (2020) Randomised controlled trial (comparator: brief telephone calls) Canada	To examine the effectiveness of the peer-based intervention on older Chinese immigrants’ psychosocial wellbeing.	A two-on-one peer-based intervention which included home visits, telephone calls, problem-solving support and community resource sharing. Volunteers (24) 8 weeks	Community (home visits, phone calls) Community-dwelling older Chinese (60)	Chinese, including Mandarin, Cantonese and other dialects Nearly all Chinese, with only one from a Southeast Asian country NR	**Primary outcomes** ***Loneliness:*** De Jong Loneliness Scale-6 ***Social support:*** Lubben Social Network scale (LSNS) ***Barriers to social participation:*** Keele Assessment of Participation **Secondary outcomes** ***Depressive symptoms:*** General Depression Scale (GDS-4) ***Anxiety:*** Chinese version of the Geriatric Anxiety Inventory-Short Form (GAI-SF) ***Life satisfaction:*** Single question—‘In general, how satisfied are you with your life?’ ***Happiness:*** Single question—‘In general, how happy do you feel?’ ***Resilience:*** Connor-Davidson Resilience Scale (CD-RISC 2) ***Purpose in life:*** Ryff’s Psychological Wellbeing Scale	When compared to the control group, the intervention group reported decrease in loneliness (1.17 [95% CI, 0.45 to 1.89], *p* = 0.018), along with an increase in resilience (1.37 [95% CI, 0.7–2.03], *p* = 0.004). No significant differences between intervention and control groups were identified for social support, barriers to social participation, depressive symptoms, anxiety, life satisfaction, happiness and purpose in life	Volunteers received training on mental health, skills to offer peer support, how to deal with grief and loss, self-help skills, stress management, goal setting, and how to build healthy relationships. Although the volunteers were unable to provide professional intervention or therapy, they actively listened to and respected the needs of the older participants. During the matching process, there were considerations made regarding the gender, age, language and education background so that some form of commonalities were linked between the older person and the peer-supporters
Miller et al. (2020) Pilot randomised controlled trial (comparator: waiting list) Lebanon	To assess the feasibility, acceptability and perceived usefulness of the intervention	Caregiver support intervention focussed on caregiver wellbeing (e.g. stress management, relaxation), drawing from a culturally integrative approach. Non-mental health specialists (NR), social worker (1) 9 weeks	Community-based organisations 151 caregivers, 79 families	Arabic Syrian (87%), Palestinian (10%), Lebanese (3%) Refugees (97%)	***Parenting:*** 24-item measure developed for the study to assess parent warmth, responsiveness and harsh parenting. ***Caregiver stress:*** 8-item scale developed for the study. ***Caregiver psychological well-being:*** Warwick-Edinburgh Mental Wellbeing Scale (WEMWBS) ***Caregiver psychological distress:*** Kessler Psychological distress (K10) ***Stress management:*** 10-item scale developed for the study. ***Child psychological wellbeing-parent report:*** Kid-KINDL	There were significant changes in the hypothesized direction on all parent-reported outcomes in the intervention group, with all *p* < 0.01. There were no significant changes on any variable in the control group	Women’s groups were co-facilitated by female facilitators. Men’s groups were co-led by male facilitators
Oh and Ell (2018) Randomised controlled trial (comparator: usual care—patient-centred medical home—referrals, care management) United States	To examine whether changes in depressive symptoms and social support prospectively predicted diabetes management amongst Hispanic patients with probable depression in patient-centred medical homes at safety-net clinics	A Helping Hand (rapport building, problem-solving, education, self-care, community resource navigation, referrals to providers) Promotora (health worker) (NR) NR	In-person and phone meetings Adults with diabetes or diabetes and cardiovascular disease or heart failure (251)	NR Hispanic NR	***Depressive symptoms:*** 9-item Patient Health Questionnaire (PHQ-9). ***Perceived social support:*** 8-item Modified Medical Outcomes Study (MOS) Social Support Survey ***Adherence to self-care:*** MOS Specific Adherence Recommendations ***Self-efficacy related to diabetes:*** Self- Efficacy for Managing Chronic Disease scale	Changes in depressive symptoms at 6- and 12-month follow-ups predicted self-efficacy and adherence to diabetes management. Changes in total social support and emotional social support were correlated with self-efficacy regarding diabetes management only at 6-month follow-up	
Poudel-Tandukar et al. (2022) Pre-post study United States	To assess the effect of a peer-led family-centred SEW intervention on preventive and mental health outcomes amongst resettled Bhutanese adults	Social and emotional wellbeing (SEW) intervention Community leaders (10) 5 weeks (90 min)	Community (home visits) Bhutanese adults (103) from 50 families	NR Bhutanese Refugees	***Depression:*** Hopkins Symptom Checklist-25 (HSCL-25) ***Anxiety:*** Hopkins Symptom Checklist-25 (HSCL-25), ***Perceived stress:*** Cohen Perceived Stress Scale (10-item version) ***Coping strategies:*** 32-item Coping Strategies Inventory-Short Form (CSI-SF) ***Social support:*** 12-item Multidimensional Scale of Perceived Social Support (MSPSS) ***Social networks:*** Lubben Social Network Scale-Revised (LSNS-R) ***Family conflict resolution:*** 17-item Family Conflict Resolution Scale (FCRS) ***Coping self-efficacy:*** 26-item coping self-efficacy (CSE)	Findings indicated a statistically significant reduction in stress, anxiety, depressive symptoms scores, and improved coping, coping self-efficacy and family and community networking scores from baseline to both follow-ups (7-day and 3-month post-intervention) (all *p*'s < 0.01). No significant changes were identified for social support or family conflict resolution	Each week, each family was provided with a package of educational materials, including leaflets containing stress management tips, strategies for strengthening coping, communication, social networking skills and pictographs on breathing exercises and yoga
Ramirez et al. (2020) Randomised controlled trial (comparator: Patient navigation only (maximum of 6 phone calls to seek information about community services, printed materials) United states	To examine the effects of enhanced patient navigation (PN) through the PN-Livestrong Cancer Navigation Services (LCNS) programme (vs PN only) on both general and disease-specific health-related quality of life in Latinx breast, prostate, and colorectal cancer survivors after primary treatment completion	PN-LCNS (promotion of use of services, help to overcome barriers to using the programme, orient participants to the availability of community resources, assist with accessing and planning future medical appointments) Patient navigators (NR) 3 months	Phone or online-based one-on-one support Hispanic/Latinx cancer survivors (288)	Spanish or English and Spanish Hispanic/Latinx NR	***Health-related quality of life (HRQOL):*** 27-item Functional Assessment of Cancer Therapy-General (FACT-G) scale ***Functional Assessment of Cancer Therapy:*** cancer-specific Functional Assessment of Cancer Therapy symptom burden subscales	PN-LCNS demonstrated a significant improvement in HRQOL in comparison with PN only for colorectal cancer survivors (males—(β, 10.074; 95% CI, 2.030–18.119; *p* = 0.014), as measured by FACT-G at 6-month follow-up; females—(β, 0.168; 95% CI, 0.030–0.305; *p* = 0.017) as measured by at 6-month follow-up; but not for breast and prostate cancer survivors *p* = 0.013)	Greater baseline HRQOL was associated with a smaller change in HRQOL from the baseline (β, − 0.321; 95% CI, − 0.397 to − 0.244; *p* < 0.001)
Weinstein et al. (2021) Randomised controlled trial (comparator: clinic-based Asthma educator [AE-C]) United states	To examine how psychosocial factors impact on asthma response to community health workers (CHW) and AE-C, as well as the impact of the interventions on psychosocial factors	CHW (e.g. education, behaviour change plans, self-management skills) CHW (2), AE-C (1) 10 visits over 12 months in their home or preferred location (e.g. school, clinic)	Home visits Children and caregivers (223 dyads)	Spanish or English Hispanic NR	***Asthma control:*** Asthma control test ***Parent mental health:*** 9-item Patient Health Questionnaire, 6-item Short Form of the PTSD Checklist—Civilian Version ***Child mental health:*** Children’s Depression Inventory 2, Patient-Reported Outcomes Measurement Information System Depressive Symptoms Parent Proxy form, Child and Parent Report of Post-traumatic Symptoms, Traumatic Events Screening Inventory- Child Report Form Revised ***Family functioning:*** Chaos, Hubbub, and Order Scale (CHAOS)	Psychosocial outcomes did not vary by group, but parent depression, parent and child PTSD symptoms, and social support showed significant improvement for both groups. CHW intervention was more effective than AE-C for improving asthma control in presence of higher baseline parent depression and PTSD symptoms (average change estimate 7.49 points; 95% CI 5.93, 9.05) versus the AE-C group (average change of 4.76 points; 95% CI 2.90, 6.63)	

^a^Where the authors presented results other than the effectiveness of interventions (e.g. demographic characteristics of the population, population needs), we have prioritised the results related to effectiveness or mediators of effectiveness and omitted other results.

*CI* confidence interval, *NR* not reported, *PTSD* post-traumatic stress disorder, *QoL* quality of life, *PROMIS* patient reported outcomes measurement information system, *RCT* randomised controlled trial, *SD* standard deviation

**Table 2:** Summary of the mixed-methods studies investigating psychosocial support programmes

Author (year) Methods used Location	Study aims	Intervention Who delivers the intervention (*n*) Intervention duration	Setting (*n*) Population (*n*)	Language Culture Migration status	Outcomes considered	Results	Comments
Blignault et al. (2022) Pre-post measures and qualitative interviews Australia	To implement and evaluate a community-based group mindfulness programme delivered to Arabic and Bangla-speaking communities. An overall programme aim was to address barriers to mental health care through supporting de-stigmatisation, assisting with coping, and facilitating access to professional care when needed	CALD Mindfulness Programme: signs of stress, helpful and unhelpful stress responses, stress management skills, breathing exercises, loving kindness, self-compassion A bilingual mental health clinician (psychologist) with support from a bilingual community worker (98)^b^ Four weeks	Online Arabic and/or Bangla speakers (44 women enrolled, 35 completed the programme)^a^	Arabic and Bangla Arabic and Bengali Migrants—most were relative newcomers under the skilled migration programme	***Pre-post measures*** ***Psychological distress:*** K10+ ***Overall experience with the program:*** “Overall, how would you rate your experience of the program?”, with participants reporting their responses on a 5-point Likert Scale ***Skills transfer:*** “Over the past four weeks, have you shared your mindfulness skills with anyone? If yes, who?” ***Qualitative interview data***	***Pre-post measures*** K10+ scores showed improvement post-programme (*p* = < 0.001). 85% of participants indicated that the effect on their overall wellbeing was ‘very good’ or ‘excellent’ and 97% rated their experience of the programme as ‘very good’ or ‘excellent’. 65.7% and 54.3% shared the acquired skills with family and friends, respectively. ***Qualitative interviews*** Feedback from participants: “I am very happy to have found this programme in my language and culture. I am able to better understand and relate to the topics”.	The programme adopted a stepped-care model for primary mental health care and a collaborative regional approach. Programme was modified in response to the COVID-19 pandemic.
Budosan et al. (2016) Survey, in-depth interviews with members of NGOs and focus groups with refugees who participated in the survey Turkey	To conduct a needs assessment of Syrian refugees in order to inform development of mental health and psychological support (MHPSS) interventions	MHPSS: vocational activities, psychological first aid and family support, social and community events, formal and informal education of Syrian children, distribution of non-food items (e.g. clothes, shoes). Psychologists, social workers and community workers (NR)^b^ NR	Community Syrian refugees (381 survey respondents; MHPSS participants—NR)	Arabic Syrian Refugees	***Surveys*** ***Humanitarian Emergency Settings Perceived Needs:*** includes a range of social, psychological and physical problem areas ***In-depth interview and focus groups data***	***Survey*** On average, participants rated 5.6 problem areas as serious. The lowest number was 0 and the highest was 21. 74% of surveyed participants rated income or livelihood as one of their three priorities. ***In-depth interviews and focus groups*** A ‘place to live in’ was a serious problem because the town is overpopulated with Syrian refugees and rents were high. Inability to acquire or pay for medication, education for children and access to drinking water were also discussed as major issues.	Other priorities included ‘clothes, shoes, bedding or blankets (24.9%)’, unequal aid distribution (24.7%) and ‘being displaced from home’ (24.1%). Implications included advocacy of temporary work permits for Syrian refugees, rent regulation, cash assistance for the most vulnerable, criteria for the distribution of humanitarian aid and establishment of a transparent and centralised information system on humanitarian aid availability.
Chemali et al. (2017) Assessment of longitudinal data, qualitative surveys Lebanon	To assess the feasibility and acceptability of an adapted version of the SMART-3RP (Stress Management Relaxation Response Resilience Training) to address the emotional and physical burden on the humanitarian field	The SMART-3RP teaches self-care strategies to protect against the negative effects of stress and improve coping skills. A Lebanese-born and Arabic speaking neuropsychiatrist (1)^b^ Four sessions	NR Social and fieldworkers (100)	Arabic Lebanese NR	***Longitudinal data (baseline and quarterly follow-ups)*** ***Stress:*** Symptom Checklist-90-Revised (SCL-90) ***Blood pressure*** ***Pulse*** ***Qualitative survey data***	***Longitudinal data (baseline and quarterly follow-ups)*** Mean SCL-90 score decreased by 14.7 ± 29.8 points (*p* < 0.0001) from baseline to follow-up 4. Mean systolic blood pressure decreased by 11.9 ± 18.4 units (*p* < 0.0001), mean diastolic blood pressure decreased by 6.4 ± 10.1 units (*p* < 0.0001) and pulse decreased by an average of 8.3 ± 15.9 units (*p* < 0.0016). ***Qualitative survey data*** Participants discussed the effect of the SMART-3RP training on stress and positivity. Many described an amelioration of anger and irritability due to mindfulness exercises, which gave them more control over stressful situations and assisted with problem-solving.	Only 52 workers completed the course. Human resources were scarce—aid workers did not have guaranteed or protected time to participate, and were often forced to choose between attending training sessions and distributing resources to refugees. Many participants voiced wishes to continue this training, and the need for mindfulness practices in their cultural context.
El-Khani et al. (2021) Quantitative measurements, interviews Serbia	***Aim 1*** To evaluate delivery and feasibility of and any potential impact of the Strong Families programme (SF) with refugees. ***Aim 2*** To assess potential benefits of SF for families, in improving family functioning, children’s psychological wellbeing and its cultural appropriateness	SF: group intervention for primary caregivers and their children that seeks to improve positive communication, ability to enforce limits, encourage good behaviour and discourage misbehaviour Facilitators who had direct access to caregivers and their children and social workers (20, of which 5 were social workers) 5 h over 3 weeks	Reception centres (3) 25 families	Serbian or Dari Afghan Refugees	***Quantitative measurements*** ***Emotional and behavioural difficulties:*** Strengths and difficulties questionnaire (SDQ) ***Parent practices, risk and parental emotional adjustment and quality of family:*** Parenting and Family Adjustment Scales (PAFAS) ***Qualitative interview data***	***Quantitative measurements*** ***Emotional and behavioural difficulties:*** SDQ scores significantly reduced before and after the programme (*t*1–*t*2; *p* = 0.004; t3, *p* = 0.002). ***Parent practices, risk and parental emotional adjustment and quality of family:*** For families who scored over the 70th percentile on each PAFAS subscale at baseline, there was a significant reduction in scores on all parenting subscales, except for parental teamwork. ***Qualitative interview data*** Themes included (i) perceived improvements in parenting practices, (ii) improved parent-child and inter-couple communication, and (iii) culturally appropriate engagement and satisfaction with SF (caregivers did not want the programme to end).	The intervention led to parents’ subjective report on reductions in child behavioural and emotional difficulties, supporting the idea that even very light touch interventions can bring about change to families in low resource challenging settings
Fine et al. (2021) Feasibility cluster randomised controlled trial (comparator: enhanced treatment as usual [ETAU] consisted of a joint adolescent and care psychoeducation session), qualitative interviews Tanzania	To evaluate the feasibility, acceptability, relevance and safety of Early Adolescent Skills for Emotions (EASE) amongst Burundian refugee young adolescents and their caregivers. Prior to implementation, EASE was adapted for use with this population to improve cultural and contextual appropriateness and acceptability	EASE contained 2 arms: (1) for adolescents—psychoeducation, stress management, behavioural activation, problem-solving and relapse prevention; (2) for caregivers—psychoeducation, active listening, slow breathing, positive parenting strategies, caregiver self-care and relapse prevention Refugees, non-specialist facilitators (5)^b^ 7 weekly group sessions (90-min each) + 2-h group session for caregivers	Refugee camp (1) 86 adolescents and 68 caregivers (numbers are discordant due to the presence of siblings in the study)	Kirundi Burundi Refugees	***RCT*** ***Adolescent-focused measures*** ***Psychological distress:*** African Youth Psychosocial Assessment (AYPA) ***PTSD symptoms:*** Child PTSD Symptom Scale (CPSS) ***Mental well-being:*** Short Warwick-Edinburgh Mental Wellbeing Scale (SWEMWBS) ***Traumatic exposures:*** Child Trauma Questionnaire (CTQ) ***Caregiver focused measures*** ***Psychological distress:*** 6-item Kessler ***Qualitative interviews***	***RCT*** ***Adolescent-focused measures*** Psychological distress significantly decreased in both the EASE group (mean change = − 6.7, *p* *<* 0.001) and the ETAU group (mean change = − 4.3, *p* = 0.02). There were no other statistically significant differences within groups. ***Caregiver focused measures*** Psychological distress decreased significantly in the EASE group (mean change = − 4.0, *p* *<* 0.001); there was no change in the ETAU group. ***Qualitative interviews*** Most adolescents and caregivers gave positive feedback on the length and frequency of EASE sessions, although several suggested that they would have benefited from more and/or longer sessions. There was a general consensus amongst participants, facilitators and school staff that EASE materials were relevant and appropriate. Most adolescents reported using strategies learned during EASE to cope with issues at home. Caregivers mentioned changes in their children, including increased initiative at home, greater discipline, feelings of wellbeing and the use of breathing exercises to feel calm. A number of caregivers also reported changes in themselves, including improved relationships with their children	Recommendations to a larger trial included (but were not limited to) targeted selection and validation of a screening tool capturing general psychological distress in this population; simplification of EASE materials to address challenges with literacy and better coordination of EASE with schools. As the authors had difficulties retaining facilitators, they highlighted the importance of monetary support and non-financial incentives (e.g. further professional development) to increase motivation and job satisfaction amongst non-specialist providers, both of which are crucial to ensuring the sustainability and scalability of task-shifting interventions
Greene et al. (2019) Desk review, consultation with key stakeholders, examination of outcome measures, interviews Tanzania	To develop an integrated health and protection intervention to reduce psychological distress and intimate partner violence (IPV), examine its relevance, acceptability and feasibility and prepare and evaluate the outcome assessment tools and research procedures	Nguvu: skills to overcome negative thoughts and self-perceptions, focussing on increasing autonomy, empowerment and strengthening linkages to community supports. Lay facilitators (2) 8 weekly sessions (2 hours each)	Refugee camp (1) Females who reported IPV (60). 40 key informants and 17 females participated in the consultation and interviews, respectively	Swahili Congolese Refugees	***Desk review*** Summary of empirical studies on mental health in the context of gender-based violence amongst Congolese women. ***Consultation*** Perspectives of stakeholders on the most common problems affecting Congolese women who experience IPV, and on available services and support. ***Outcome measures*** ***IPV:*** Abuse Assessment Screen ***Psychological distress:*** Hopkins Symptom Checklist ***Anxiety and depression:*** Harvard Trauma Questionnaire ***Qualitative interview data***	***Desk review and consultation*** Both confirmed that IPV was prevalent amongst Congolese women, but very few interventions have been evaluated to address mental health and IPV in this population. ***Outcome measures*** Overall, outcome measures revealed good test-retest reliability and internal consistency. Some items were weakly correlated with the remaining items and perhaps unrelated to functioning (e.g. farming, trading and other income-generating activities). ***Qualitative interview data*** The intervention was thought to be relevant and helpful. The most difficult parts of Nguvu to understand were the ABCs (becoming aware of the connection between an event, the resulting thought and how this thought makes the person feel) and changing thoughts.	Participants recommended that the age composition of intervention groups be homogenized to avoid having relatives of different generations within the same group, which can result in relational power dynamics that may inhibit group discussion
Jacquez et al. (2019) Pre-post measurements, field notes United States	To identify stressors experienced by Latino immigrants and goals of stress reduction identified by them. To determine if a Community-based participatory research (CBPR) stress intervention delivered by peers could engage Latinx immigrants and improve stress management and perceived support	CBPR (active listening, identify strategies to cope with stress, promote SMART goal setting) Co-researchers (17)^b^ Three weekly sessions (1st session—88 min; 2nd session—64 min and 3rd session 67 min)	NR Latinx immigrants (116)	Spanish Latinx Immigrants in new migration cities, with a large majority of participants (81%) being undocumented	***Pre-post measurements*** ***Perceived stress:*** Perceived stress scale ***Social support:*** 8-item measures from PROMIS, PROMIS Emotional support and PROMIS informational support ***Stress management:*** Patient Activation measure ***Psychological resilience:*** Brief resilience scale ***Qualitative data from field notes*** ***Stressors and goals:*** documented on field notes	***Pre-post measurements*** Emotional Support *t*(111) = − 2.44, *p* = 0.016, informational support *t*(110) = − 3.023, *p* = 0.003, and stress management *t*(110) = − 5.966, *p* = 0.000 improved significantly. Resilience and perceived stress did not change. ***Stressors and goals:*** Family, children, work, health, immigration and spouse were the most commonly reported stressors. Goals were mostly related to spending time with family and friends, exercising, walking, listening to music and eating healthy	Most (76%) of the sample did not graduate from high school and 81% reported household incomes under $25,000. Traditional stress reduction programmes tend to focus on cognitive strategies or relaxation, yet 78% of Latinx immigrants chose to engage in physical activities, such as taking a walk or exercising for stress reduction.

^a^Although the paper focuses on the evaluation of the online group programmes rolled out during the pandemic, this programme has attracted a total of 489 participants between March 2017 and September 2021.

^b^The people who delivered the intervention had the same cultural background and/or linguistic background as clients.

*DACA* deferred action for childhood arrivals, *NGOs* non-governmental organization, *SMART* specific, measurable, achievable, relevant and time-bound.

**Table 3:** Summary of qualitative studies investigating psychosocial support programmes

Author (year) Study Design Location	Study aims	Intervention Who delivers the intervention (*n*) Intervention duration	Setting Population (*n*)	Language Culture Migration status	Study findings	Comments
Amodeo et al. (2004) Programme evaluation drawing on case examples and questionnaires United States	To describe the culturally specific programme elements, early accomplishments, barriers and changes to goals and objectives of the intervention: Project Sangkim	Assessment and weekly treatment services for substance abuse for Cambodian adults including counselling, home visits and family involvement, acupuncture and coordination with community services A co-therapy team including an English-speaking social worker (1) and Cambodian non-clinician substance abuse case manager (1) Varied	Mental Health Centre Cambodian adults with alcoholism (*n* = ‘small client base’)	Khmer Khmer Migrants and Refugees	1. Education on addiction as a treatable illness is needed; 2. Treatment programmes should be situated in non-stigmatizing settings; 3. Addiction and domestic violence treatment should be combined; 4. Clients with chronic substance abuse will likely be the first referred; 5. Full-time programmes are preferred due to severity and clients’ auxiliary needs; 6. Dual diagnoses of substance abuse and other mental disorders (e.g. PTSD) are common; 7. Subcontracts with other refugee support organisations can support referrals; 8. Nonverbal treatments (e.g. acupuncture) should be considered; 9. Capacity building of refugee community members should be prioritised; 10. Culturally appropriate alternatives to in-patient residential treatment should be considered	Providing Project Sangkim through a mental health centre increased connotations to stigma, posing a barrier to uptake Limited budgets often limit a programme’s capacity to document their approach. Documentation, research and evaluation are necessary to support knowledge development of what approaches work
Barudy (1989) Programme model explication Belgium	To describe the central ideas that support a medical-psychosocial support programme for Latin American Political Refugees	Latin American Collective of Psychosocial Work (COLAT; 1976–1985) and EXIL for refugees outside of Latin America (from 1985) Clinicians (psychologists and social workers) and community allies (inferred) Ongoing (inferred)	Mental health programme Political Refugees (4315)	Spanish, Portuguese & other (inferred) Mainly Chilean, Latin American Refugees	Identity challenges are central to being a political refugee, being exiled from one’s country due to acts of political resistance. Community action is central to addressing such identity challenges. Thus, their programme includes 1. Self-help groups to denounce political violence, create new rituals and community; 2. Clinical support: a quick diagnoses followed by the care required, ranging from medical assistance, to family therapy, psychotherapy, group therapy, and hospitalisation; 3. Support to facilitate reintegration into work and society.	Community is positioned as essential to countering the trauma experienced by political refugees. Reconstructing a sense of community is necessary to identity reconstruction. Limited detail on the programme is provided; the role of community allies is not clear
Behnia (2007) Descriptive exploratory drawing on questionnaires Canada, USA, Australia and England	To identify challenges that organizations face in recruiting and retaining befrienders; to learn strategies used to overcome barriers	Befriending programmes 25 organisations Varied	Communities Volunteer befrienders of refugees	Varied (Befriender: English, French, other) (Befriender: Western, refugee background) Refugees (Befriender: Citizens)	Befrienders are mainly educated white women, who assist with advocacy and activities of daily life (e.g. shopping, computer skills, job seeking, refugee hearings). Recruiting a more gender and ethnically diverse pool of befriender volunteers was said to be difficult. Key barriers to recruitment included limited resources preventing broader advertising or capacity to convert voluntary to paid arrangements; liability of professionals working as volunteers; time constraints of work and family; negative stereotypes about refugees; perception of the volunteer work’s effectiveness; fear of the daunting nature of the work. Key challenges to retention included: limited resources, seasonal fluctuation in volunteers’ availability, cultural, political and language differences between refugees and volunteers, reluctance to trust amongst some refugees. Targeted advertising and public presentations were used to overcome recruitment challenges. Training, support, following-up and matching volunteers and refugees based on gender, age and interests were key strategies for overcoming retention barriers	The authors recommend involving refugees and befrienders in the planning and overseeing of befriending programmes; investigating both refugees’ and befrienders’ perceptions of their experiences, and collecting socio-demographic data on refugees and volunteers.
Burns et al. (2019) Programme review United States	To understand the transformation of the programme from an academic-community partnership to a community-led programme, and review the parent education programme for immigrant families from Mexico	Safe, Secure and Loved (SSL): a mindfulness-based, trauma-informed parent education programme delivered by promotoras to Latino communities to support early childhood resilience and nurturing parenting (e.g. mindfulness and self-compassion exercises) Promotoras (female volunteer paraprofessional health and education advisors) Varied	Community, with support from a large non-profit multiservice agency (10 groups for parents of babies in year 5; 12 in year 6) Economically disadvantaged Latino families in San Jose, California (< 100 families in year 5 of the programme; 150 families in year 6)	Mainly Spanish (inferred) Latinx; Mexican Immigrant families	Drawing on online surveys and in-person interviews with the academic researcher, agency education director, agency staff and promotoras from the community workforce, authors identified a process of 1. identifying shared goals across the researcher and director before exploring a researcher partnership and laying the conceptual groundwork; 2. Exploring resourcing options and fielding interest preceding promotoras experiences of connection and personalisation, which were central to the installation phase; 3. Implementing feedback loops and leadership—sustained through mutuality and trust—to address challenges; 4. Reinforcing feedback loops and coaching to manage change and growth, supported by promotion of a staff member to lead the community workforce. Authors attribute their success to the high value of the topic of healthy parenting; the success of mindfulness and trauma-based approaches at addressing stressors related to poverty; the importance of the community workforce in culturally contextualising the learning.	Rigorous research is needed to (1) demonstrate the benefits of such programmes to, potentially, reducing child abuse and neglect and reorienting protective systems towards initiative supported by a community workforce; (2) explore the impact of the community work on promotora, allowing for a better understanding of what motivates them towards this community engagement
Fietz and Stupp (2019) Evaluation: content analysis of 2 focus groups with Turkish seniors Germany	To assess whether native language groups promote social participation of Turkish seniors	Native language groups hosted by a German organisation Community organisation: ZWAR (NR) NR	Community-based organisations Turkish seniors	Turkish Turkish Migrants	Social participation of Turkish seniors was improved through regular meetings with other Turkish seniors, shared understanding, mutual emotional support and further opportunities to participate in community events. Turkish seniors described improved comfort in socialising and increased self-confidence	The organisation of inter-ethnic cultural events fostered a unifying group identity amongst Turkish seniors and followed a strengths-based approach. Traditional gender roles were described as a barrier to equal participation
Hassan (2013) Personal Reflection Syria	To describe the experience of a Syrian psychologist supervising an outreach programme as part of the UN Refugee Agency in Syria	Psychosocial Outreach Volunteer Project, with weekly activities Peer volunteers NR	Refugee camp in Syria NR	Arabic Iraqi Refugee	The psychosocial outreach volunteer programme draws volunteers from the local refugee community with relevant professional training or skills (e.g. social work or yoga). The volunteers are provided with training. Services include those aimed at providing psychosocial support and community activities: drama, art, sewing, music, cooking, yoga, first aid, coffee, peer support and education. Participants and volunteers all benefit. The activities create meaning, self-worth and relationship building	Key challenges are the cost of support activities and access. Many refugees experiencing mental health disorders refuse support
Im and Rosenberg (2016) Qualitative evaluation United States	To assess the impact of a pilot peer-led community health workshop for Bhutanese refugees	Peer-led community health workshops on healthy eating, resettlement stressors, coping, psychological distress and mental health Peer community health worker, after 4 days of training 8 sessions over 2 months	Community Bhutanese adults (27)	Nepali Bhutanese Refugees	Drawing on focus group discussions, health promotion outcomes, health practices, emotional health, sense of community and belonging were all found to improve following the intervention	The peer-led community health workshops facilitated social capital development
Khalsa et al. (2020) Semi-structured interviews, informed by phenomenological approach United states	To explore the experiences of service providers who work with refugee communities in the Northern Colorado area, especially related to acculturation, compassion fatigue and burnout	Refugee service organisation provides support with education (e.g. on culture and language), programme administration (e.g. programme design, staffing, management), and community navigation (e.g. accessing healthcare, education, housing, employment training) Educators, administrators and community navigators, with the latter being members of the community they serve 3 months	In-person (inferred) Refugees living in Northern Colorado (360)	Varied Central America, Middle East, Africa, Southeast Asia Refugees	Drawing on analysis of 60-minute interviews with seven service providers, service providers reported that clients often feel there are cultural and language walls that inhibit their access to services and the broader community. Compassion fatigue and vicarious trauma are common in this work, but limited time and support for self-care were exacerbated by the energy administrators expended on seeking further funding for the service. For community navigators (who were from the communities they serve), boundaries between work and home were difficult to maintain, further limiting time for self-care	The political climate in the US in 2019–2020 was noted as being less hospitable to refugees and immigrants, culminating in reduced funding and job security, placing further strain on financial and emotional resources. Large variations in education—with refugees ranging from having advanced tertiary qualifications to illiterate—also posed challenges
Makhoul et al. (2012) Qualitative Interviews Lebanon	To describe the experience of and assess the benefits to Youth Mentors supporting the Qaderoon mental health intervention for youths in a Palestinian refugee camp in Lebanon	Qaderoon—a theory-based youth mental health intervention project for 10–14-year-olds within a Palestinian refugee camp—involved 45 skill-building sessions in communication, problem-solving, self-esteem and self-responsibility 6 university level facilitators with experience working with youth; 23 17–25-year-old Palestinian Youth Mentors living in or near the refugee camp 1 year: 2008–2009	Refugee camp Youths (18) and their family and friends (10) within a 1.6 km^2^ refugee camp containing 14,000 to 18,000 residents	Arabic (inferred) Palestinian Refugees	Findings draw on thematic analysis of interviews with Youth Mentors and their family and friends. Incentives for joining included the income, exposure to social and community work, and the opportunity to interact with Palestinian children. Reasons for continuing with the programme included the sense of accomplishment and importance to the programme, as well the programme’s impact on their skill development. Several noted their improved confidence in speaking with more educated individuals, and emotion regulation in managing anger	Recency effect and desirability bias could have influenced interviews, with youth mentors and families emphasising the positive influence of the programme out of a desire for the programme to continue
McFarlane and Fehir (1994) Documentation of MM programme using qualitative interviews and descriptive data United States	To examine the programme Madres a Madres (MM) and report feedback results	MM seeks to increase access to prenatal care by providing information regarding healthcare, shelter, food, child care, legal aid and employment to pregnant women. Volunteer mothers (NR) NR	Community At the Fifth year of the programme, 551 pregnant women were given information about various issues (e.g. prenatal care, housing, food pantries)	Spanish Hispanic NR	Drawing on broad informal qualitative interview data with stakeholders and extensive interviews with 5 MM volunteers, the programme was found to improve communication amongst community residents, form coalitions to solve the community’s problems, increase personal development of programme’s staff and volunteers. Women reported that the programme enhanced their quality of life, self-esteem and increased a sense of unity	The MM programme emphasizes health as a continuous process rather than a terminal event which patriarchal models cast as a “goal.”; Rather than a hierarchical power structure where responsibilities are subdivided and difficult to identify, women in the MM programme enjoy the “power of unity”: the group shares responsibility for decisions and action in the community
Msengi et al. (2015) Descriptive case study using focus groups, observations and evaluation forms United States	To describe the Women of Care Project	A single multicultural women’s programme facilitated in the Midwest state of Iowa including a support group, conversational partner matching, home visits and other community events (e.g. International Tea Party) A programme founder of African descent ran the programme with two translators and two university-student interns One meeting per month over one year, plus other activities	Community and home Immigrant women (15 per meeting on average)	Varied Varied Immigrants and refugees	Findings suggest the programme—featuring a support group and activities such as cooking tea socials and stress management sessions—fostered improvement in wellbeing that extended beyond the individual to participants children and extended families. The support helped women to overcome barriers in language, culture, poverty and discrimination and function in their new communities. Connection with others with similar migration histories helped to alleviate isolation	Study limitations not included
Orpinas et al. (2020) Heuristic inquiry of programme outcomes United States	To describe the challenges faced by promotoras and propose solutions	A community-based participatory research programme—Lazos Hispanos—for Latinx community members, aiming to bridge access to social and health care services Promotora volunteers (9), after 78 hours of training One year (inferred)	Community, Southeastern USA Promotoras (5)	Spanish (inferred) Latinx	Drawing on two group interviews, promotoras describe challenges related to competing family and promotora work priorities, gendered power differences in working with men, emotional impact of participants’ problems—especially their lack of hope, English language barriers, ethnocentrism and discrimination amongst some providers, transportation, setting boundaries and the burden of data collection	Nine promotoras were initially involved, but four left the programme at the end of the first year due to family or work commitments
Paloma et al. (2020) Process evaluation drawing on peer support group transcripts, evaluations, online comments and fieldnotes Spain	To analyse refugees’ processes of resilience and empowerment during participation in a community-based intervention	Across two phases of a community-based intervention, refugees first received and received training in delivering group-based peer support, and second delivering peer group support Phase 1: researcher; Phase 2: newly trained cultural peer support group facilitators 2–3 h × 2 session/week over 15 weeks	Community 10 adult refugees (6 men; 4 women)	Spanish, Ukranian, French Varied Refugees	Following training to become peer support group facilitators—in guided relaxation, individual reflection, sharing migrant stories, presenting community resources and identifying personal strengths—participants described increased resilience, empowerment and self-efficacy	First programme to sequentially combine peer support and peer mentoring formats. Participants may not be representative of the wide population, as they were selected for their leadership skills and sensitivity to social engagement
Perez et al. (2015) Photovoice methodology to reflect on factors affecting mental health United States	To describe the perspectives of Latina promotoras and community forum attendees regarding challenges to mental health for Latinas, and potential interventions	Amigas Latinas Motivando el Alma: a community-partnered research project to identify symptoms and improve Latinas’ mental health outcomes through promotoras and advocacy Promotoras NR	Community, North Carolina Promotoras (8) and community members (146)	English and Spanish Latinx (Mexican, South American) Immigrants	From photovoice discussions with promotoras, challenges affecting mental health were identified: isolation of transitioning to life in the US, feeling stretched between wanting to prioritise culture and involvement in parenting and long work hours, structural and social barriers to education for Latinas and children, and discrimination against undocumented migrants related to housing and obtaining a drivers’ licence. Increasing community awareness of the challenges to mental health and information about resources in the community were key recommendations.	Help-seeking was described as a taboo for Latinas Photovoice was found to be an effective tool for including the experiences of Latinas with limited English proficiency in research to inform interventions
Priebe et al. (2012) Expert interviews 14 European countries	To identify good practice in providing mental health care to socially marginalised groups	Strategy for organising the provision of mental health care to socially marginalised groups Clinical and non-clinical staff NR	Community & clinic Experts in psychiatry, psychology, social work, occupational therapy, nursing, medicine, community work, law, social science and social policy (154)	Varied European Citizens, refugees, people seeking asylum and irregular migrants	Drawing on expert interviews, four components of good practice were identified for providing mental health care to the long-term unemployed, street sex workers, homeless, refugees/people seeking asylum, irregular migrants and travelling communities: outreach programmes; access to multifaceted physical and mental health services to reduce referrals; coordination across services; raising awareness of services to marginalised groups and practitioners	Findings may be specific to highly deprived urban areas. Understanding of context-specific factors was limited
Ruiz-Sánchez et al. (2021) Qualitative retrospective interviews United States	To examine the perspectives of participants in De la Mano con la Salud and explore their perceptions of emotional, information and instrumental support provided	De la Mano con la Salud (lend a hand to health): a community-based participatory project offering non-directive social support by helping Latino immigrant men to articulate their goals (using the Wheel of Life) and find solutions to the problems they prioritise, such as connecting to health and social services and the larger community Latino immigrant male promotores (community health workers), with training Monthly in-person meetings, with additional phone meetings, over 6 months	New and emerging Latinx communities in Pennsylvania Latino immigrant men (182)	Spanish (inferred) Latinx (primarily Mexican, 113, but also Central American, 62) Immigrants	Drawing on findings from 23 interviews, Latino men described a need to have their identity recognised through engagement with their ethnic community. They found the intervention effective in addressing their health, legal and work needs, while also promoting a feeling of ‘respaldo’: perceived social support important to health.	Intervention is offered at an individual level, thus cannot resolve issues requiring intervention at a structural level
Quosh (2011) Evaluation, including qualitative and quantitative needs assessment, knowledge tests standardised training evaluation of trainers. Syria	Description and evaluation of a training programme to build capacity in delivering psychosocial support	Takamol (meaning integration in Arabic)—a UNHCR pilot programme aiming to support refugee mental health through (1) capacity building (training) of frontline workers in schools, clinics, humanitarian aid programmes and universities, (2) case management of people most at risk, and (3) an outreach volunteer programme. Psychiatrists and Psychologists deliver the training to frontline workers 170 h of training over 1 year	Community Trainers (44), with 40 completing the training	Arabic, Kurdish (inferred) Iraqi Refugees	All trainers passed the knowledge tests. Standardised training evaluations suggested the training supported high knowledge absorption, strong ability to run training and consistency in delivery. Attendance ranged from 100 to 87.5%. It was predicted that the capacity building would eventually lead to improved access to psychosocial support and wellbeing amongst Iraqi refugees	Findings support the cascade approach. High staff turnover and differences in interagency politics, timelines and incentives posed challenges. Findings were limited to the effectiveness of the training; further research on the impact of the capacity building on service users is needed
Schmid (2019) Interviews with volunteers Germany	To provide a practical and empirical example of a care-oriented approach to integration	Refugee support work guided by an ethics of care approach to integration Female volunteers from the majority population Varied	Community refugee support organisations in two large cities and smaller towns Female refugee support work volunteers (22)	German	Drawing from literature on the ethics of care to interpret findings from interviews with German female refugee support volunteers, their work involved relationship building, attentiveness, responsibility, empathy, respecting difference and respect (to counter power differences). Volunteers saw their work as political and useful in working towards inclusive societal change	Research with service users is needed. It is important to note the inherent possibility of unequal power relations associated with an ethics of care
Wei et al. (2021) Historical case study and interviews Australia	To describe and review the Victorian Foundation for Survivors of Torture Community Liaison Worker (CLW) role in delivering trauma-informed community capacity building	Trauma-informed community capacity building to support: acculturation, social network building, service engagement and the adoption of meaningful roles in Australian society CLWs (7) Intervention is ongoing; research duration: 12 months	Community, Victoria Community members who are survivors of torture (4308)—permanent residents, people seeking asylum or temporary visa holders		Drawing on a thematic analysis of interviews with 7 CLWs, it was found that CLWs have diverse and multifaceted dual roles as community leaders and employees. Their roles involve working with clients to meet short-term goals, while also working to achieve community empowerment. Specifically, this involves (1) supporting refugees by improving their access to services and helping them to develop independence; (2) supporting service providers by improving their cultural capabilities; (3) developing community self-sufficiency through recognising and addressing a community’s needs	Resourcing and awareness raising were identified as barriers, with underfunding and poor recognition of the complexity of the CLW compromising capacity building. Peer support and supervision were identified as key enablers
Wells et al. (2018) Grounded theory, using interviews to inform transactional concepts Jordan	To develop and test an ecological model of adaptation to displacement, relevant to the experiences of Syrian refugees	Psychosocial organisations supporting the Syrian refugee community in Jordan. NR NR	Community, Amman Syrian (many of whom had recently been displaced) and Jordanian key informants (29), working in relevant psychosocial organisations	Arabic (inferred) Syrian Refugees	Findings, based on interviews with key informants, suggest the Arabic concepts of Karama (dignity) and Sudme (emotional impact of the crisis) were central to the adaptation of an ecological model to the Syrian refugee context. Karame was described as central to identity in relation to family, self, social standing and culture. Sudme suggested the emotional responses to potentially traumatic events was normal. Overall, their emotional responses to trauma (sudme) were compounded by the stress of being in a new community, where one’s dignity tied to one’s identity (karame) was displaced	The ecological model—underscoring the importance of environment, and change in environment as central to understanding refugees’ values and behaviours—was found to be appropriately adapted using the Syrian concepts of wellbeing (Karama, Sudme). An intersectional theoretical lens is suggested to further recognise the importance of gender, class, sexuality and ethnicity as intersecting identity positions
Wiles et al. (2019) Qualitative interviews with service users, non-users, volunteers and stakeholders New Zealand	To explore culturally diverse older adults’ attitudes to and experiences of befriending services in Aotearoa (New Zealand)	Age Concern New Zealand ‘Accredited Visiting Service’—a befriending service for older people, that aims to provide supportive contact, and improve health and wellbeing Paid staff (30) and 2600 volunteers 1-hour weekly	Community Culturally diverse older adults (2500)	English, Mandarin, Korean European, Māori, Pacific, Chinese, Korean	Findings are based on thematic analysis of interviews. Befriending services alleviate social isolation and loneliness. Barriers to access include feeling undeserving (others need it more) and knowledge of and appropriateness of service. Ideal befriending services were said to support real and reciprocal relationships, foster access to community and connection to culture, and be reliable	Cultural alignment—through engagement with culturally specific organisations—should be a priority in improving uptake amongst culturally diverse older adults. Service providers were present in some interviews, which may have influenced findings

*NR* not reported, *CLW* community Liaison worker

## Theoretical Underpinnings

Our review draws from a critical realist framework and Brossard and Chandler’s (2022) taxonomy of positions on culture and mental health. The critical realist framework combines a realist ontology (there is something real to find out about) with a relativist epistemology (knowledge is influenced by several factors [e.g. culture, language] and people will come to know things in different ways) (Stutchbury, 2022). It foregrounds the relational nature of sociocultural and contextual forces underpinning conceptualisations, practice delivery and outcomes (Pawson & Tilley, 2005), while considering how mechanisms of support are affected by and intersect with such forces to arrive at intended and unintended outcomes (Mathison, 2005).

During analysis, we drew on explication and examples from Brossard and Chandler’s explication of their taxonomy (explicated in the introduction) to categorise each intervention as adopting one positions—universalist, split-relativist or radical—based on how mental health disorders and interventions were conceptualised. For instance, if an intervention was conceived in a global north cultural context, based on Euro-American conceptualisations of mental health and delivered to CALD participants with little to no changes, this was categorised as adopting a universalistic position. Interventions using accompanying outcome measures developed in Euro-American contexts were similarly categorised as universalistic, or split-relativist if translated for the local context. If, in contrast, an intervention was developed by or in collaboration with CALD participants, drawing on culturally specific or non-western conceptualisations and/or outcome measures, this was categorised as adopting a radical relativist position.

## Data synthesis

**Fig. 2:**
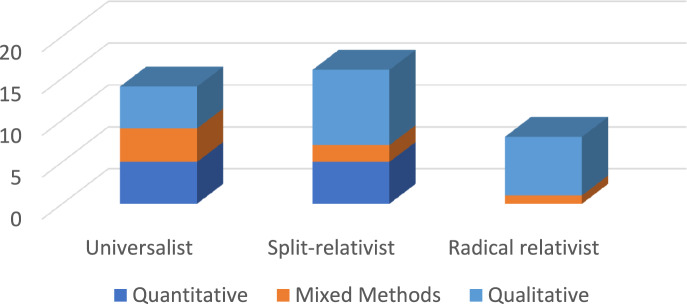
Interventions mapped against Brossard and Chandler’s (2022) taxonomy

The data synthesis of included studies was twofold. First, we used the data extracted to describe such studies, and we present such description in both textual and tabular forms (see Tables 1, 2 and 3). Results from our synthesis by methods and thematic insights prompted us to engage more critically. Thus, second, we critically assessed the literature drawing from Brossard and Chandler’s (2022) taxonomy. Here, assumptions underpinning conceptualisations of intersections across culture and mental health were analysed, along with power differences and epistemological positioning. This critical analysis is presented in narrative form, organised by methods and thematic insights. This structure is in conversation with Brossard and Chandler’s taxonomy (see Fig. 2) and consideration of the methodological orientation of the included studies,with, for example, no quantitative studies fitting into the radical relativist category. As the purpose of the present review was to provide an overview of existing evidence regardless of the methodological quality of included studies, we did not conduct a critical appraisal of included studies.

## Results

A total of 4851 studies were identified. After removing duplicates (*n* = 464) and screening titles and abstracts, 38 studies met the inclusion criteria: 10 quantitative, 7 mixed-methods and 21 qualitative studies (see Fig. 1).

The study designs and research methods varied, with a majority using interviews, focus groups, surveys and questionnaires. Most studies were conducted in North America (i.e. United States of America *n* = 17; Canada *n* = 2) or Europe (i.e. Germany *n* = 3, Belgium *n* = 2, Spain *n* = 2), with a minority conducted in Australia, Tanzania and Lebanon (*n* = 3 each). Study participants included service users (typically migrants and refugees), as well as service providers and LHWs (also known as community health workers, peer support workers and ‘promotoras/es’ [Spanish term for support workers]). Including multiple stakeholders within study designs—particularly within qualitative studies—acknowledged the complexity of the services and interventions under investigation, with several employing a cascade model where service users progress through training and experience to become service providers. Most studies (*n* = 30) were recent, published after 2014. Given the high number of studies from North America and the political unrest in Syria over the past decade, it is not surprising that most included studies—where a country of origin for service users was specified—cited migrants, refugees and people seeking asylum as coming from Mexico and Syria. This increased scholarly interest in establishing the merits of non-clinical approaches to supporting CALD populations’ mental health reflects growing interest in non-clinical approaches, such as those using LHWs (Barnett et al., 2021).

Our critical approach to reviewing existing scholarship allows us to prioritise context *and* draw on theory. Specifically, we move beyond the synthesis found in traditional systematic reviews to critically analyse (Grant & Booth, 2009) differing approaches to conceptualising, programming and evaluating non-clinical psychosocial support for migrant populations from CALD backgrounds. We start with the overarching findings from our critical rapid review. In the sub-sections that follow, we provide an overview of the key findings from publications included within the critical rapid review, categorised by study type: quantitative, mixed-methods and qualitative. We then apply Brossard and Chandler’s (2022) taxonomy of approaches to culture and mental illness, fostering a theory-informed critical assessment of the state of the literature. Finally, we respond directly to our research question in the discussion, offering cumulative reflections informing our conclusions on non-clinical psychosocial mental health support for CALD populations.

## Quantitative Studies: Favouring Behavioural and Cognitive Approaches Over Social and Political Aspects

Ten quantitative studies were included (Badger et al., 2020; Cardeli et al., 2020; Greene et al., 2021; Im et al., 2018; Lai et al., 2020; Miller et al., 2020; Oh & Ell, 2018; Poudel-Tandukar et al., 2022; Ramirez et al., 2020; Weinstein et al., 2021), all published in the last decade (see Table 1). Most quantitative study designs were randomised controlled trials (RCTs) (*n* = 6) (Badger et al., 2020; Lai et al., 2020; Miller et al., 2020; Oh & Ell, 2018; Ramirez et al., 2020; Weinstein et al., 2021) followed by three pre/post studies (Cardeli et al., 2020; Im et al., 2018; Poudel-Tandukar et al., 2022) and one pilot RCT (Greene et al., 2021). The studies often targeted specific populations (e.g. Bhutanese (Cardeli et al., 2020), Congolese (Greene et al., 2021) or Somali refugees (Im et al., 2018)). Interventions varied greatly, including trauma-systems therapy skills-based groups (Cardeli et al., 2020), a violence- and mental health-focussed group intervention (Greene et al., 2021), trauma-informed psychoeducation (Im et al., 2018), peer support (Lai et al., 2020), wellbeing focussed interventions (Miller et al., 2020; Poudel-Tandukar et al., 2022), health system navigation support (Oh & Ell, 2018; Ramirez et al., 2020) and support from community health workers (Weinstein et al., 2021). Likewise, the study aims varied from—for example—evaluating the impact of skills-based groups (Cardeli et al., 2020), to examining whether changes in depressive symptoms and social support prospectively predicted diabetes management (Oh & Ell, 2018).

In most studies (*n* = 8), interventions were delivered by one or more peers from a similar cultural background (Badger et al., 2020; Greene et al., 2021; Im et al., 2018; Lai et al., 2020; Oh & Ell, 2018; Poudel-Tandukar et al., 2022; Ramirez et al., 2020; Weinstein et al., 2021). Two studies involved the participation of clinician and cultural broker simultaneously (Cardeli et al., 2020; Miller et al., 2020). Intervention length varied from five (Poudel-Tandukar et al., 2022) to 12 sessions (Cardeli et al., 2020; Im et al., 2018; Ramirez et al., 2020; Weinstein et al., 2021) and the settings included school (Cardeli et al., 2020), community groups (Greene et al., 2021; Im et al., 2018; Miller et al., 2020), home visits (Poudel-Tandukar et al., 2022; Weinstein et al., 2021), phone/online support (Badger et al., 2020; Ramirez et al., 2020) or both in-person meetings and phone support (Lai et al., 2020; Oh & Ell, 2018). Thirty-five distinct outcomes were considered across the ten studies, with depression (Badger et al., 2020; Cardeli et al., 2020; Greene et al., 2021; Lai et al., 2020; Oh & Ell, 2018; Poudel-Tandukar et al., 2022), anxiety (Greene et al., 2021; Lai et al., 2020; Poudel-Tandukar et al., 2022), post-traumatic stress (Cardeli et al., 2020; Greene et al., 2021; Im et al., 2018) being the most common outcomes. Amongst the studies which investigated the effectiveness of interventions, most identified significant improvement for at least one outcome (Badger et al., 2020; Im et al., 2018; Lai et al., 2020; Oh & Ell, 2018; Poudel-Tandukar et al., 2022; Ramirez et al., 2020; Weinstein et al., 2021), with two exceptions (Cardeli et al., 2020; Greene et al., 2021).

About the studies in this thematic grouping, we can make two critical observations. First, attrition is often a problem in this type of research. Drop-out rates (reported in seven of the ten studies) were above 20% for most studies (Badger et al., 2020; Im et al., 2018; Miller et al., 2020; Oh & Ell, 2018), potentially due to reasons associated with the very impetus for seeking support: resettlement, insecurity and migration. For instance, in one study (Im et al., 2018), only 58% of the 250 recruited participants completed the whole intervention (12 sessions). Second, there is a notable misalignment between interventions and noted causes of mental illness in quantitative studies. Interventions were all underpinned either by behavioural or cognitive approaches, focussing on individual changes. Yet, many authors underscored the social and political causes of service users’ poor wellbeing—what Marmot (2004) refers to as the ‘causes of the causes’ of poor health, or the social determinants of health (Marmot, 2004). In one paper (Miller et al., 2020), for example, the authors mention that 75% of Syrian refugees live below the poverty line in Tripoli (where the study was conducted) on less than USD$120 per month. It was also reported that their living conditions were precarious and work restrictions were increasingly stringent. Despite this, the intervention focussed only on relaxation and breathing exercises, not on access to income or meeting basic needs.

## Mixed-Methods Studies: Considering Cultural Aspects when Developing Interventions and Moving Towards Advocacy

Seven mixed-methods studies were included (see Table 2). Studies drew from a range of quantitative and qualitative methods, including pre-post measures (Blignault et al., 2022; El-Khani et al., 2021; Fine et al., 2021; Jacquez et al., 2019), interviews (Blignault et al., 2022; Budosan et al., 2016; El-Khani et al., 2021; Fine et al., 2021; Greene et al., 2019), desk reviews (Greene et al., 2019; Schmid, 2019), surveys (Budosan et al., 2016; Chemali et al., 2017), consultation with stakeholders (Greene et al., 2019), field notes (Jacquez et al., 2019) and assessment of longitudinal data (Chemali et al., 2017). The aims also varied across the seven studies, ranging from informing intervention implementation and development (Blignault et al., 2022; Budosan et al., 2016; Greene et al., 2021), to feasibility and acceptability (Chemali et al., 2017; El-Khani et al., 2021; Fine et al., 2021; Greene et al., 2019), and impact and benefit (El-Khani et al., 2021; Jacquez et al., 2019). All mixed-methods studies targeted a particular population (e.g. people who speak Arabic and/or Bengali (Blignault et al., 2022), social and field workers at a refugee camp (Chemali et al., 2017), people from refugee backgrounds (Budosan et al., 2016; El-Khani et al., 2021; Fine et al., 2021; Greene et al., 2019) and Latinx immigrants (Jacquez et al., 2019)), rather than offering interventions for broad populations. Six interventions were very specific [e.g. mindfulness (Blignault et al., 2022), stress management (Chemali et al., 2017), parental support (El-Khani et al., 2021), psychological support (Fine et al., 2021; Jacquez et al., 2019), family violence-focussed (Greene et al., 2019)]. One offered a broad intervention, which included the distribution of non-food items (Budosan et al., 2016).

Intervention length varied from three (El-Khani et al., 2021) to eight sessions (Greene et al., 2019). Most interventions (*n* = 5) were provided by people from the same cultural background as participants (Blignault et al., 2022; Budosan et al., 2016; Chemali et al., 2017; Fine et al., 2021; Jacquez et al., 2019). For two of the interventions, information about the cultural and linguistic background of facilitators was not clearly reported (El-Khani et al., 2021; Greene et al., 2019). Over half (*n* = 4) of the interventions involved clinicians as facilitators, supported by a bilingual support worker (Blignault et al., 2022; Budosan et al., 2016; El-Khani et al., 2021) or alone (Chemali et al., 2017).

The quantitative outcome measures considered in the studies included psychological distress (Blignault et al., 2022; Chemali et al., 2017; Fine et al., 2021; Greene et al., 2019; Jacquez et al., 2019), perceived humanitarian emergency needs (Budosan et al., 2016), blood pressure and pulse (Chemali et al., 2017), emotional and behavioural difficulties (El-Khani et al., 2021), parenting practices, risk and parental emotional adjustment and family quality (El-Khani et al., 2021), PTSD symptoms (Fine et al., 2021), mental health and wellbeing (Fine et al., 2021), traumatic exposures (Fine et al., 2021), family violence (Greene et al., 2019), depression and anxiety (Greene et al., 2019), social support (Jacquez et al., 2019), psychological resilience (Jacquez et al., 2019) and stress management (Jacquez et al., 2019). Five of the seven mixed-methods studies included pre- and post-measures (i.e. four studies compared pre and post data within the same group and one between the intervention and control groups) (Blignault et al., 2022; Chemali et al., 2017; El-Khani et al., 2021; Fine et al., 2021; Jacquez et al., 2019). Amongst these, four of them identified significant differences in the outcomes of interest after receiving the intervention (i.e. pre- and post-comparisons within the same group) (Blignault et al., 2022; Chemali et al., 2017; El-Khani et al., 2021; Jacquez et al., 2019) and one study identified no significant differences between intervention and control groups (Fine et al., 2021). Qualitative findings often enabled authors to better understand participants’ experiences with the intervention (Blignault et al., 2022; Chemali et al., 2017; El-Khani et al., 2021; Fine et al., 2021; Greene et al., 2019), as well as their needs (Budosan et al., 2016; Jacquez et al., 2019).

We drew several critical observations from the review of studies in this thematic grouping. First, interventions may have had aims beyond individual improvement. Authors of one study explicitly used their data to engage in advocacy work, seeking action from institutions and government (Budosan et al., 2016). Second, all studies that compared pre- and post-intervention data reported a significant improvement in at least one outcome and were relatively short, indicating that ‘light touch’ interventions can have impact with these populations. Third, culture should inform intervention design. Jacquez et al. (2019) found Latinx immigrants preferred to engage in physical activity as a form of relaxation, as opposed to more common forms of relaxation (e.g. mindfulness activities) evaluated in other research. This suggests that, rather than a universalistic approach, culturally appropriate care recognises the person, and their cultural identity, and tailors the intervention accordingly (Jacquez et al., 2019). Fourth, improved resourcing and professional development may help to ameliorate issues of high staff turnover. Reflecting on their difficulties in retaining facilitators, Fine et al. (2021) highlight the importance of monetary support and non-financial incentives (e.g. further professional development) to increased motivation and job satisfaction amongst non-specialist providers, both of which are crucial in ensuring sustainability and scalability (Fine et al., 2021).

## Qualitative Studies: Overcoming Systemic Barriers Through Consideration of Stakeholders’ Perspectives and Context

A total of 21 qualitative studies were included (see Table 3). Sampling ranged from small (e.g. *n* = 1–7 focus groups, interviews or reflections Fietz & Stupp, 2019; Hassan, 2013; Khalsa et al., 2020)) to extensive [e.g. thousands of clinical case notes (Barudy, 1989), 20–30 (Im & Rosenberg, 2016; Ruiz-Sánchez et al., 2021; Schmid, 2019; Wells et al., 2018) up to 100+ interviews (Wiles et al., 2019)]. Some studies had limited engagement with service users, with a key focus being volunteers’ (Burns et al., 2019; Hassan, 2013; Khalsa et al., 2020; Orpinas et al., 2020; Schmid, 2019; Wei et al., 2021) or experts’ (Priebe et al., 2012; Wells et al., 2018) experiences. One study included the perspectives of service users and non-users (Wiles et al., 2019). The programmes under investigation varied in their focus, ranging from specific to broad in terms of issues, interventions and populations.

Many services were provided by people from the same cultural or refugee backgrounds as clients (Hassan, 2013; Im & Rosenberg, 2016; Makhoul et al., 2012; McFarlane & Fehir, 1994; Orpinas et al., 2020; Paloma et al., 2020; Perez et al., 2015; Ruiz-Sánchez et al., 2021; Wei et al., 2021; Wiles et al., 2019). Others were mixed with, for example, some trainers (Quosh, 2011) and workers (Wells et al., 2018) coming from the target refugee population; or with self-help and support groups run by and for refugees combined with other activities offered by staff, often using translators (Barudy, 1989; Msengi et al., 2015). Several programmes—particularly those based in Germany, the United States and across multiple countries—were delivered by non-clinical service providers from the majority population—i.e. providers not considered CALD in each country (Amodeo et al., 2004; Behnia, 2007; Burns et al., 2019; Fietz & Stupp, 2019; Khalsa et al., 2020; Priebe et al., 2012; Schmid, 2019).

Engagement with theory-informed models of service varied, with some not engaging with any (Wiles et al., 2019), and others centring such models. These models/theories included process model framework (Quosh, 2011); biopsychosocial model of identity (Barudy, 1989); strength-based approach (Msengi et al., 2015); trauma-informed approach (Wei et al., 2021); intersectionality theory (Orpinas et al., 2020); Praxis, Empowerment, Awareness, Consensus and Evolvement (PEACE) (McFarlane & Fehir, 1994); social capital (Im & Rosenberg, 2016); ethics of care (Schmid, 2019); and an ecological model (Makhoul et al., 2012; Wei et al., 2021; Wells et al., 2018).

Drawing from the studies included within this grouping, we make the following observations. First, barriers to accessing mainstream services were commonly reported. Specific cultural groups regarded the experience of mental illness as highly stigmatising (Amodeo et al., 2004), and crisis experiences associated with being a refugee often provoked mistrust in authorities, including mainstream health care professionals (Barudy, 1989). Second, a ‘cascade approach’, where professionals learn to become trainers and then train non-clinician service providers—often from the same background (e.g. language, culture, migrant/refugee experience) as clients—was valued. This cascade approach not only addresses gaps in service availability and appropriateness, but empowers the target population (Quosh, 2011). Third, precarious resourcing was said to exacerbate commonly faced challenges related to high staff and volunteer turnover, access to adequate spaces and large disparities in meeting service users’ needs (Behnia, 2007; Hassan, 2013; Im & Rosenberg, 2016; Khalsa et al., 2020; Wei et al., 2021; Wiles et al., 2019). Fourth, intervention models that foreground considerations of context, culture, community, relationality and power were found to be important to countering barriers to support and enhancing empowerment (McFarlane & Fehir, 1994). Fifth, documentation, research and evaluation of outcomes were described as rare and challenging, with many volunteers finding the process of collecting data burdensome, culturally inappropriate and/or outside the scope of their work (Amodeo et al., 2004; Orpinas et al., 2020). Sixth, the importance of service users’ perspectives was highlighted repeatedly, especially in studies with limited representation of service users in their samples (Burns et al., 2019; Hassan, 2013; Khalsa et al., 2020; Orpinas et al., 2020; Schmid, 2019; Wei et al., 2021).

## A Critical Assessment of the State of the Literature Based on Included Studies

We draw on Brossard and Chandler’s (2022) taxonomy of approaches to culture and mental illness, and well-known anthropological and sociological work on the social determinants of health (Behnia, 2007) and critical approaches to global mental health (Beneduce, 2019), to offer a theory-informed critique of existing scholarship on non-clinical psychosocial support for CALD service users. To start, we mapped interventions against Brossard and Chandler’s (2022) taxonomy (see Fig. 2).

**Fig. 3:**
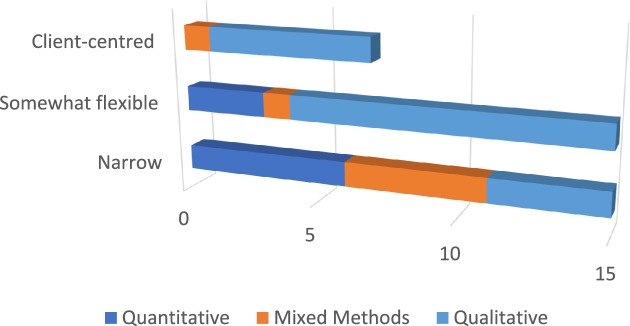
Scope of service

Fourteen studies adopted a universalist framework that risks ‘reinforc[ing] the idea that whiteness is ordinary’ (Chandra, 2021, p. 771) and being underpinned by ‘Western hegemonic values concerning health, illness, and healing’ (Beneduce, 2019, p. 710), especially quantitative and mixed-methods studies (Badger et al., 2020; Behnia, 2007; Blignault et al., 2022; Cardeli et al., 2020; Chemali et al., 2017; El-Khani et al., 2021; Fine et al., 2021; Khalsa et al., 2020; Paloma et al., 2020; Poudel-Tandukar et al., 2022; Priebe et al., 2012; Quosh, 2011; Ramirez et al., 2020; Weinstein et al., 2021). In such studies, the intervention focus tended to be narrow and individualistic, and measured impact was more often limited to psychological outcome measures (see Table 4). Eight studies were on the opposite end of the taxonomy spectrum, and could be characterised as working within a radical relativist framework (e.g. Budosan et al., 2016; Fietz & Stupp, 2019; Hassan, 2013; McFarlane & Fehir, 1994; Msengi et al., 2015; Perez et al., 2015; Ruiz-Sánchez et al., 2021; Wei et al., 2021). These studies tended to be of programmes offering culturally safe and client-centred services (see Fig. 3).

**Table 4:** Psychological outcome measures

Psychological concept	Scales
Depression	Patient-Reported Outcome Measures (PROMIS)
	Depression Self-Rating Scale for Children (DSRS-C)
	Hopkins Symptom Checklist (HSCL-25)
	General Depression Scale (GDS-4)
	9-Item Patient Health Questionnaire (PHQ-9)
	Child's Depression Inventory 2
	Harvard Trauma Questionnaire
Stress & Distress	Kessler Psychological Distress (K10)
	General Symptom Distress Scale
	Cohen Perceived Stress Scale (10-item version)
	Symptom Checklist-90-Revised (SCL-90)
	Blood Pressure
	Hopkins Symptom Checklist
	Perceived Stress Scale
	Patient Activation Measure
Anxiety	Hopkins Symptom Checklist 25 (HSCL-25)
	Geriatric Anxiety Inventory, Short Form (GAI-SF)
Trauma & PTSD	War Trauma Screening Scale (WTSS)
	UCLA PTSD Reaction Index
	Harvard Trauma Questionnaire (HTQ)
	Trauma Exposure Scale
	PTSD Check List—Civilian Version (PCL-C)
	Child and Parent Report of PT Symptoms, Traumatic Events Screening Inventory—Child Report Form Revised
	Child PTSD Symptom Scale (CPSS)
Coping	32-item Coping Strategies Inventory-Short Form (CSI-SF)
	26-item coping self-efficacy (CSE)
Resilience	Connor-Davidson Resilience Scale (CD-RISC 2)
	Brief Resilience Scale
Wellbeing	Warwick-Edinburgh Mental Wellbeing Scale (WEMWBS)
	Short Warwick-Edinburgh Mental Wellbeing Scale (SWEMWBS)
	27-item Functional Assessment of Cancer Therapy-General (FACT-G)

Such programmes worked to identify the social determinants of health exacerbating a clients’ and communities’ poor mental health. Empowerment, not just treatment, was often a stated objective of these services. The imposition of Euro-American outcome measures was avoided. Instead, impact tended to be measured experientially—relative to the clients’ goals—and/or using broad outcome measures such as the Humanitarian Emergency Settings Perceived Needs Scale, which includes measures of social, psychological and physical challenges (Budosan et al., 2016).

Most (*n* = 16) studies were in the middle—working within a split-relativist framework—often focussed on interventions aimed at helping clients navigate the Eurocentricity and complexity of mainstream services (Amodeo et al., 2004; Barudy, 1989; Burns et al., 2019; Greene et al., 2019, 2021; Im & Rosenberg, 2016; Im et al., 2018; Jacquez et al., 2019; Lai et al., 2020; Makhoul et al., 2012; Miller et al., 2020; Oh & Ell, 2018; Orpinas et al., 2020; Schmid, 2019; Wells et al., 2018; Wiles et al., 2019). Such services were often flexible in scope and delivered by trained peers, such as LHWs, peer support workers, or promotoras/es. Measures of impact—aligned with a cascade model—tended to prioritise the experiences of all involved stakeholders (i.e. service users, service providers and LHW). When scales were employed, psychosocial outcomes were more often measured.

## Discussion

We summarise our findings and offer cumulative reflections guided by the research question to inform our conclusions on non-clinical psychosocial mental health support for CALD populations. This critical rapid review identified 38 studies that report on non-clinical psychosocial mental health support for CALD populations. The focus of interventions evaluated in these studies varied greatly—from narrow and individualistic to broad and systemic, beyond individual improvement, steering towards advocacy and addressing social determinants of health. Nonetheless, all quantitative and mixed-methods studies but one reported improvement in at least one of the outcomes of interest when these compared pre- and post-intervention data. In qualitative and mixed-methods studies, consideration of the experiences and perspectives of CALD populations and other relevant stakeholders provided insights not only into the impact of the interventions investigated, but also the complexity of needs, suggesting that their involvement in the intervention and/or research process is critical. For instance, service user input brought to the fore many important barriers to accessing mainstream mental health services, including the cultural disconnection in how mental health is conceptualised across Euro-American cultures and the difficulties of navigating a myriad of health, welfare, legal and non-government services. Where the perspectives of LHWs were considered in qualitative studies, other important barriers were identified, such as culturally inappropriate outcome measures that hindered the evaluation of interventions and the need for comprehensive resourcing to prevent staff attrition such as sufficient financial support, considerations of vicarious trauma and staff development opportunities.

Drawing from both our descriptive and critical analysis of included studies, we identified features for *designing* and *delivering* non-clinical psychosocial support services for CALD clients. We elucidate these below under two headings, and in Box 1.

**Box 1:** Features for non-clinical psychosocial support services for CALD immigrant clients

Design
– Recognises the eurocentrism of universalist frameworks, and counters this by foregrounding culture in intervention design, working from a **culturally relativist position**
– Works from an appropriate **model** (e.g. the ecological model) that centres clients, flexibility, context, culture, community and relationality
– Foregrounds **advocacy**, **empowerment** and the **social determinants of health** in delivering culturally appropriate psychological *and* social interventions
Setting
– Uses a **community** setting; the stigma associated with mental health care settings can prevent engagement
Delivery
– Engages **staff** from a **similar background to clients,** fostering the trust, rapport and understanding necessary to make psychosocial support accessible, appropriate and effective
– Adopts a **cascade model**, empowering members of the community being served – through training – to become lay-health workers
Evaluation
– Uses **broad evaluation** measures, beyond psychometric scales, including service providers’ and service users’ perspectives
Funding
– **Resourcing is adequate and** ongoing, increasing opportunities for professional development and reducing challenges with staff turnover, spacing and the diversity of clients’ needs

### Designing Psychosocial Support Services for CALD Populations

When designing services for CALD populations, the eurocentrism of universalist frameworks should be recognised (Beneduce, 2019; Chandra, 2021), even when operating within the ethnopsychiatric paradigm. These frameworks may be well-meaning, in the way they attempt to ‘render immigrants ‘legible’ to the state’ (Sangaramoorthy & Carney, 2021, p. 592) so that they and their needs may be classified and served within biomedical and bureaucratic structures under which healthcare is provided and, in some cases, paid for. Yet by conceptualising and shaping CALD populations’ mental health experiences to fit a particular universal model, inevitably some forms of suffering are validated, some misinterpreted and others ignored entirely.

Recognizing the limitations of such frameworks helps to counter them by foregrounding culture in the intervention design and working from a culturally relativist position (Brossard & Chandler, 2022). Furthermore, prioritising advocacy, empowerment and the social determinants of health in delivering culturally appropriate non-clinical psychological and social interventions seemed to reduce the misalignment between the interventions and causes of poor mental health.

This aligns well with the philosophy of cultural humility, which upholds self-reflexivity, appreciation of lay expertise, power-sharing and continuous learning from patients by health and social care providers (Tervalon & Murray-Garcia, 1998). Co-production of psychosocial support services with CALD community stakeholders and those with lived experience may provide a framework for centring these priorities in service design, and co-production has been shown to increase the sustainability of health and social care innovations (Overton et al., 2024). However, it is important that researchers and healthcare practitioners remain mindful of the intense stigma around mental health in many CALD communities (Lam et al., 2010). Such stigma not only hampers access to, and utilization of, mental health care services, but can also dissuade community stakeholders from wishing to engage in co-design of solutions lest their involvement reify a problem deliberately ignored or denied by the community (Amann & Sleigh, 2021).

### Delivering Psychosocial Support Services for CALD Populations

Amongst the identified practices, our analysis recognised five points as noteworthy. First, the use of a community setting for the service may help engage CALD populations in service use, as the stigma associated with mental health care settings can prevent engagement. Locating within the social setting of the community can also help increase awareness of the social and socioeconomic atmosphere in which members of communities are living and working—what Farmer (2004, p. 308) might call the ‘materiality of the social’. Such awareness may reduce the risk of service providers interpreting social problems as ‘emotional’ or ‘psychological’ and treating them inappropriately (Sangaramoorthy & Carney, 2021).

Second, engaging staff from a similar background to clients can help foster the trust, rapport and understanding necessary to make non-clinical psychosocial support accessible, appropriate and effective. Experiences of a different health system in the home country may influence the patients’ expectations of the host country’s healthcare system they attend and health professionals they see (Majumder et al., 2015; O'Donnell et al., 2008). If these expectations are left unacknowledged or unaddressed, a lack of confidence and trust in the health professional may weaken the effectiveness of a health consultation (O'Donnell et al., 2008). Ethnic, cultural and linguistic congruity between patients and healthcare professionals has been shown to be beneficial to patient experiences, (Martin et al., 2019) outcomes (Greenwood et al., 2020) and trust in services (LaVeist et al., 2003). However, it is important that an intersectional approach be taken (Crenshaw, 1991), whereby staff and patients alike are not reduced solely to the singular identity facet of their ethnicity or language spoken, and additional factors which may shape the care relationship, such as gender, faith, age, experience of migration and discrimination are equally considered in terms of their influence on the care relationship.

Third, our findings highlight the efficacy of a cascade model, which empowers members of the community being served—through training—to become LHWs. The benefits of involvement in state and community services, for example through volunteering, for CALD community members have been demonstrated, including increased social integration, counteracting stereotypes and providing an anchor for generating and reifying a sense of belonging and relevance in place (Ambrosini & Artero, 2023; Haas, 2013). Such involvement, described by Ambrosini and Artero (2023) as ‘citizenship from below’, can also serve to legitimise the place of CALD communities as stakeholders within the state health service, with a right to effective treatment.

Fourth, we emphasise that the use of broad evaluation measures, beyond psychometric scales, and including service providers and service users’ perspectives, is more likely to provide meaningful insights into the feasibility and effectiveness of services. Drawing from lessons learned in international development and evaluation of development programmes, an evaluative approach drawing on anthropological principles may provide a more holistic, critical assessment not only of the service but also of any indirect and unexpected impacts it may have. Harrison (2015) points to a ‘politics of knowledge production’ within development and of how ‘particular disciplinary perspectives may come to dominate’ decisions on whether or not an intervention worked. This is particularly relevant to the healthcare setting where ‘quality improvement’ and implementation science paradigms dominate (Cribb et al., 2023) but an anthropological (and particularly medical anthropological) epistemology may enrich understanding.

Finally, we stress the need for commissioning bodies to provide resources and funding on an ongoing basis, both as a means to increase opportunities for professional development and to reduce challenges with staff turnover, physical space and the diversity of clients’ needs. Admittedly, healthcare services globally are increasingly institutionally and politically driven to ensure value-for-money, particularly tax-payer money (World Health Organisation, 2021). Yet as Farmer (2004, p. 313) points out ‘this ideology is indebted to and helps to replicate inequalities of power’, where those most in need of resources and funding to overcome health inequalities are least likely to receive it, and least likely to show ‘return-on-investment’ in ways which are purely fiscally measurable.

### Contextualising Findings—Implications for Research and Policy

CALD populations often face health inequities, which can be exacerbated by health systems that make it harder for them to access necessary care (Matlin et al., 2018). For instance, culturally inappropriate care, costs of services, lack of interpreters, lack of familiarity with the health system of the adoptive country, cultural influences on healthcare seeking behaviour, coping and presentation of symptoms often make access to mental health services more difficult for individuals from CALD backgrounds compared to the general population (Long et al., 2021; Pottie et al., 2011; Shannon et al., 2015; Ziersch et al., 2020). This may be interpreted as a form of structural violence (Farmer, 2004; Galtung, 1969). Therefore, it is unsurprising that people from CALD populations are less likely to seek care for mental health concerns (Alonso et al., 2007; The WHO World Mental Health Survey Consortium, 2004), or to be referred to care effectively by primary care providers (Kapadia et al., 2022). Intersectoral action—where LHWs act as service brokers—is likely necessary to address health, economic and social policies that hinder this population’s access to psychosocial support and healthcare more broadly. Likewise, multi-sectoral responses could affect social forces such as food security and discrimination, which in turn influence (mental) health outcomes.

Relatedly, there have been calls to achieve health equity through upstream action on the social determinants of health (WHO, 2011; Marmot et al., 2008). Pauly et al. (2019) argue that reducing health inequities is a social, ethical and economic imperative, requiring a reassessment of how healthcare is approached and a restructure of systems to purposefully address such determinants (Pauly et al., 2019). We concur and expand on their argument by proposing that non-clinical psychosocial services can assist this undertaking by addressing structural injustices through actions on social forces that impact CALD populations, furthering an appreciation of mental health as more than cognitive: as biosocial, sociocultural and intimately connected to oppression (Farmer, 2004; Galtung, 1969). Our findings suggest that in addition to connecting individuals to service providers, LHWs can do so through advocacy, education programmes, enhancing community networks, providing health system navigation support, reducing food insecurity and more. Inclusion of non-clinical psychosocial support in healthcare systems may improve reach and sustainability of services for CALD populations. Within this context, strategies to involve service users in the planning and implementation of non-clinical psychosocial support services are warranted. Culture should inform intervention design, as should an ethos of cultural humility. Involving community members from the early stages of intervention planning and service delivery can ensure these are relevant (Vaughn et al., 2017), improve the rigour of research and facilitate policy change (Wallerstein, 2020) and intervention sustainability (Overton et al., 2024). That said, as individuals from CALD populations have criticised previous research for not providing information in a sensitive manner that could have better informed them before data collection (Dyregrov et al., 2000), it is important to consider that trust issues may be exacerbated if participants from CALD populations have a history of trauma, concerns about privacy and/or visa status (Enticott et al., 2017), either directly or historically (Fassin & Rechtman, 2005). Future interventions and studies should consider these potential barriers when involving communities and potential service users across different stages of planning and evaluation.

Based on our findings, we identify areas requiring further investigation in order to better inform community services and public health policy. For instance, future research could consider prioritising theory-informed psychosocial interventions that look beyond individualistic understandings of mental illness and sees these as embedded in structures, systems, politics and cultures. Likewise, the results presented here prompt us to encourage future studies to draw on and foreground considerations related to the Brossard and Chandler’s (2022) positionings, which would likely encourage a shift in research and practice away from the epistemic imposition of universalistic approaches towards the cultural safety of relativism.

### Strengths and Limitations

To the best of our knowledge, this is the first critical rapid review of scholarship on non-clinical psychosocial support for CALD populations. We considered studies of all designs and conducted in any country, with relevance for the comprehensiveness of our findings. We conducted this review using well-established methods (Grant & Booth, 2009), frameworks (Pawson & Tilley, 2005)**,** interdisciplinary theoretical frameworks and taxonomies (Brossard & Chandler, 2022). Notably, the team’s cultural diversity (including being CALD) and mix of experiences add to the comprehensiveness of our analysis and findings. The critical approach to the review is also a strength, offering theory-informed as well as methodological platforms for scrutiny and knowledge production. Yet, all approaches to research are partial, and this critical rapid review is no exception. Specifically, the temporally constrained nature of a rapid approach to reviewing means we could not take an exhaustive approach to searching, and therefore, grey literature was excluded. Furthermore, our searching—while inclusive of several languages—was limited to the databases to which we had access. It is likely that relevant interventions for CALD populations were not covered here, potentially due to services’ lack of resources to cover costs of assessing outcomes and producing publications. Nonetheless, the considerable number of records identified—initially and for final inclusion—testifies to the breadth of the search. Finally, the variation in terms to describe CALD populations is high (e.g. migrants, refugees, immigrants, minorities, asylum seekers), and while we attempted to make our search inclusive, the inconsistencies in terminology may mean that some potentially eligible studies were missed.

## Conclusion

Our results indicate that the potential of non-clinical interventions delivered by LHWs is hindered by precarious resourcing, culturally inappropriate outcome measures and limited consideration of service-users’ and staff’s perspectives. We identified avenues for overcoming these barriers when designing psychosocial support services for and with CALD populations—recognising the eurocentrism of universalist frameworks, working from a split-relativist or culturally relativist position and prioritising the social determinants of health. For researchers, these results also demonstrate the merits of adopting Brossard and Chandler’s (2022) typology in working with and evaluating non-clinical psychosocial support for immigrants from CALD backgrounds. We recommend that in future studies, researchers work beyond dominant individualistic and universalistic understandings of mental ill-health. Drawing on our results, we invite policymakers to use community settings, engage staff from similar backgrounds to clients, empower community members to become LHWs, provide resources and use broad evaluation measures when delivering interventions and services to CALD populations. Enacting these recommendations is likely to improve the reach, cultural safety and impact of interventions and services, ultimately reducing inequities.

## Supplementary Information

Below is the link to the electronic supplementary material.

Supplementary file1 (DOCX 15 KB)

Supplementary file2 (DOCX 32 KB)

## Footnotes

1 Here, we use the term psychosocial support services to refer to services that involve elements of non-clinical support informed by psychology, social work and sociology for people who present with mental health challenges such as anxiety and depression.

2 By critical we refer here to a questioning of mainstream Euro-centric conceptualisations of mental health, supported by our theoretical underpinnings which emphasise context and relativity over the epistemic imposition of universalism.
