# Supplementary material for: Non-clinical Psychosocial Mental Health Support Programmes for People with Diverse Language and Cultural Backgrounds: A Critical Rapid Review
Source: Cult Med Psychiatry. 2025 Jan 29;49(3):585–648. doi: 10.1007/s11013-024-09893-1 (PMC12374882; doi:10.1007/s11013-024-09893-1)
Supplement: Supplementary file 1 — Supplementary file1 (DOCX 15 KB) [file 11013_2024_9893_MOESM1_ESM.docx]

| Concept | Search Terms | Subject headings (MeSH) |
| --- | --- | --- |
| Populations with diverse language and cultural backgrounds | culturally diverse OR linguistically diverse OR migrant OR refugee OR asylum seeker OR ethnic | PubMed/PsycInfo: “linguistics” OR “culture” “transients and migrants” OR “refugees” OR “ethnicity” OR “ethnology”  Sociological Abstracts: “EH” (ethnology) |
| Non-clinical psychosocial mental health support programs | Focus | |
|  | AND (non-clinical AND “mental health”) OR (non-medical AND “mental health”) OR psychosocial OR “psycho-educational skill building” OR psycho-education OR integration OR advocacy OR engagement | PubMed/PsycInfo: “mental health” |
|  | Setting | |
|  | AND community OR community service OR drop-in service OR peer support OR support OR group-based counselling OR social prescribing | PubMed/PsycInfo: “residence characteristics” OR “social welfare” OR “counseling” OR “social behavior” OR “socialization” OR “health” AND “occupational groups” |
|  | Who delivers | |
|  | AND health worker OR support aid OR peer-led OR volunteer OR cultural commission | PubMed/PsycInfo: “volunteers” OR “culture” OR “ethnology” |

Appendix A. Critical Rapid Review Search Strategy
